# Genome mining of the citrus pathogen *Elsinoë fawcettii*; prediction and prioritisation of candidate effectors, cell wall degrading enzymes and secondary metabolite gene clusters

**DOI:** 10.1371/journal.pone.0227396

**Published:** 2020-05-29

**Authors:** Sarah Jeffress, Kiruba Arun-Chinnappa, Ben Stodart, Niloofar Vaghefi, Yu Pei Tan, Gavin Ash

**Affiliations:** 1 Centre for Crop Health, Institute for Life Sciences and the Environment, Research and Innovation Division, University of Southern Queensland, Toowoomba, QLD, Australia; 2 Graham Centre for Agricultural Innovation, (Charles Sturt University and NSW Department of Primary Industries), School of Agricultural and Wine Sciences, Charles Sturt University, Wagga Wagga, NSW, Australia; 3 Department of Agriculture and Fisheries, Queensland Government, Brisbane, QLD, Australia; University of Nebraska-Lincoln, UNITED STATES

## Abstract

*Elsinoë fawcettii*, a necrotrophic fungal pathogen, causes citrus scab on numerous citrus varieties around the world. Known pathotypes of *E*. *fawcettii* are based on host range; additionally, cryptic pathotypes have been reported and more novel pathotypes are thought to exist. *E*. *fawcettii* produces elsinochrome, a non-host selective toxin which contributes to virulence. However, the mechanisms involved in potential pathogen-host interactions occurring prior to the production of elsinochrome are unknown, yet the host-specificity observed among pathotypes suggests a reliance upon such mechanisms. In this study we have generated a whole genome sequencing project for *E*. *fawcettii*, producing an annotated draft assembly 26.01 Mb in size, with 10,080 predicted gene models and low (0.37%) coverage of transposable elements. A small proportion of the assembly showed evidence of AT-rich regions, potentially indicating genomic regions with increased plasticity. Using a variety of computational tools, we mined the *E*. *fawcettii* genome for potential virulence genes as candidates for future investigation. A total of 1,280 secreted proteins and 276 candidate effectors were predicted and compared to those of other necrotrophic (*Botrytis cinerea*, *Parastagonospora nodorum*, *Pyrenophora tritici-repentis*, *Sclerotinia sclerotiorum* and *Zymoseptoria tritici*), hemibiotrophic (*Leptosphaeria maculans*, *Magnaporthe oryzae*, *Rhynchosporium commune* and *Verticillium dahliae*) and biotrophic (*Ustilago maydis*) plant pathogens. Genomic and proteomic features of known fungal effectors were analysed and used to guide the prioritisation of 120 candidate effectors of *E*. *fawcettii*. Additionally, 378 carbohydrate-active enzymes were predicted and analysed for likely secretion and sequence similarity with known virulence genes. Furthermore, secondary metabolite prediction indicated nine additional genes potentially involved in the elsinochrome biosynthesis gene cluster than previously described. A further 21 secondary metabolite clusters were predicted, some with similarity to known toxin producing gene clusters. The candidate virulence genes predicted in this study provide a comprehensive resource for future experimental investigation into the pathogenesis of *E*. *fawcettii*.

## Introduction

*Elsinoë fawcettii* Bitancourt & Jenkins, a necrotrophic fungal species within the Ascomycota phylum (class Dothideomycetes, subclass Dothideomycetidae, order Myriangiales), is a filamentous phytopathogen which causes a necrotic disease, known as citrus scab, to the leaves and fruit of a variety of citrus crops around the world. Susceptible citrus varieties include lemon (*Citrus limon*), rough lemon (*C*. *jambhiri*), sour orange (*C*. *aurantium*), Rangpur lime (*C*. *limonia*), Temple and Murcott tangors (*C*. *sinensis* x *C*. *reticulata*), Satsuma mandarin (*C*. *unshiu*), grapefruit (*C*. *paradisi*), Cleopatra mandarin (*C*. *reshni*), clementine (*C*. *clementina*), yuzu (*C*. *junos*), kinkoji (*C*. *obovoidea*), pomelo (*C*. *grandis*) and Jiangjinsuanju (*C*. *sunki*) [1–9]. Numerous pathotypes of *E*. *fawcettii* are defined by host range, including the Florida Broad Host Range (FBHR), Florida Narrow Host Range (FNHR), Tyron’s, Lemon, Jinguel, SRGC and SM, while cryptic and novel pathotypes are also reported [1, 3, 10]. Only the Tyron’s pathotype (which infects Eureka lemon, Rough lemon, clementine, Rangpur lime and Cleopatra mandarin) and the Lemon pathotype (which only infects Eureka lemon, Rough lemon, Rangpur lime) have been described in Australia [2, 3, 7], however *E*. *fawcettii* has reportedly been isolated from kumquat (*Fortunella* sp.), tea plant (*Camellia sinensis*) and mango (*Mangifera indica*) [11], indicating a wider range of pathotypes to be present in Australia. Additional species of *Elsinoë* found causing disease in Australia include *E*. *ampelina*, which causes anthracnose to grapes [12] and two *E*. *australis* pathotypes; one which causes scab disease to jojoba (*Simmondsia chinensis*) [13] and a second found on rare occasions on finger lime (*C*. *australasica*) in Queensland forest areas [14]. Species of *Elsinoë* causing crop disease in countries neighbouring Australia include *E*. *batatas*, which causes large yield losses in sweet potato crops in Papua New Guinea [15, 16] and *E*. *pyri*, which infects apples in organic orchards in New Zealand [17]. Around the world there are reportedly 75 *Elsinoë* species, the majority of which appear to be host specific [18]. While citrus scab is not thought to affect yield, it reduces the value of affected fruit on the fresh market. Australia is known for producing high quality citrus fruits for local consumption and export, and so understandably, there is great interest in protecting this valuable commodity from disease.

*Elsinoë fawcettii* is commonly described as an anamorph, reproducing asexually. Hyaline and spindle shaped conidia are produced from the centre of necrotic citrus scab lesions [19, 20]. Conidia are dispersed by water splash, requiring temperatures between 23.5–27°C with four hours of water contact for effective host infection. Therefore, disease is favoured by warm weather with overhead watering systems or rain [21]. Only young plant tissues are vulnerable to infection; leaves are susceptible from first shoots through to half expanded and similarly fruit for 6 to 8 weeks after petal fall, while mature plants are resistant to disease [19]. Cuticle, epidermal cells and mesophyll tissue are degraded within 1 to 2 days of inoculation, hyphal colonisation proceeds and within 3 to 4 days symptoms are visible [20, 22]. After formation of necrotic scab lesions on fruit, twigs and leaves, conidia are produced from the scab pustules providing inoculum for further spread. Within 5 days, host cell walls become lignified separating infected regions from healthy cells, which is thought to limit internal spread of the pathogen [20]. The necrosis that occurs during infection is produced in response to elsinochrome, a well-known secondary metabolite (SM) of species of *Elsinoë*. Elsinochromes are red or orange pigments which can be produced in culture [23, 24]. In aerobic and light-activated conditions, reactive oxygen species are produced in response to elsinochromes in a non-host selective manner, generating an environment of cellular toxicity [25]. Elsinochrome production is required for full virulence of *E*. *fawcettii*, specifically the *EfPKS1* and *TSF1* genes are vital within the elsinochrome gene cluster [26, 27]. However, two points indicate that *E*. *fawcettii* pathogenesis is more complex than simply the result of necrotic toxin production: (I) the production of elsinochrome appears to be variable and does not correlate with virulence [28]; and (II) elsinochrome is a non-host selective toxin, yet *Elsinoë* species and *E*. *fawcettii* pathotypes cause disease in a host specific manner. Host-specific virulence factors targeted for interaction with distinct host proteins to overcome immune defences, prior to elsinochrome production, could explain the observed host specificity. Candidate virulence genes may include effectors and cell wall degrading enzymes. Effectors are secreted pathogen proteins, targeted to either the host cytoplasm or apoplast, which enable the pathogen to evade recognition receptor activities of the host’s defence system and, if successful, infection proceeds. Resistant hosts, however, recognise pathogen effectors using resistant (R) genes which elicit plant effector-triggered immunity and pathogenesis is unsuccessful [29, 30]. While it was previously thought that necrotrophic fungal pathogens would use only a repertoire of carbohydrate-active enzymes (CAZymes) or SMs to infect host plants [31], there is increased awareness of their utilisation of secreted protein effectors [32–37], highlighting the importance of protein effector identification in all fungal pathogens. Frequently shared features of effectors include; a signal peptide at the N-terminal and no transmembrane helices or glycosylphosphatidylinositol (GPI) anchors. Other features less frequently shared include; small size, cysteine rich, amino acid polymorphism, repetitive regions, gene duplication, no conserved protein domains, coding sequence found nearby to transposable elements, and absence in non-pathogenic strains [38–45]. Furthermore, some appear to be unique to a species for example the necrosis-inducing protein effectors NIP1, NIP2 and NIP3 of *Rhynchosporium commune* [46] and three avirulence effectors AvrLm1, AvrLm6 and AvrLm4-7 of *Leptosphaeria maculans* [47]. Others have orthologous genes or similar domains in numerous species for example the chorismate mutase effector, Cmu1, of *Ustilago maydis* [48] and the cell death-inducing effector, MoCDIP4, of *Magnaporthe oryzae* [49]. Understandably, with such a large variety of potential features, effector identification remains challenging. Effectors are found in biotrophs, for example *U*. *maydis* [50–53], hemibiotrophs, such as *L*. *maculans* [54–56], *M*. *oryzae* [57, 58], *R*. *commune* [46] and *Verticillium dahliae* [59–61], necrotrophs, for example *Botrytis cinerea* [62, 63], *Parastagonospora nodorum* [34, 42, 64], *Pyrenophora tritici-repentis* [65], *Sclerotinia sclerotiorum* [32] and also the hemibiotroph/latent necrotroph *Zymoseptoria tritici* [66]. Genomic location has potential to be an identifying feature of virulence genes in some species, for example pathogenicity-related genes of *L*. *maculans*, including those coding for secreted proteins and genes potentially involved in SM biosynthesis, are found at higher rates in AT-rich genomic regions in comparison to GC-equilibrated blocks [47]. It is thought that effectors and their target host proteins co-evolve, in a constant arms race [67], presenting genomic regions with higher levels of plasticity as potential niches which harbour effector genes.

Another group of virulence factors likely to play a role in *E*. *fawcettii* pathogenesis are cell wall degrading enzymes (CWDE), these are CAZymes, including glycoside hydrolases, polysaccharide lyases and carbohydrate esterases, which can be secreted from fungal pathogens and promote cleavage of plant cell wall components [68–70]. Cell wall components, such as cellulose, hemicelluloses (xyloglucan and arabinoxylan) and pectin (rhamnogalacturonan I, homogalacturonan, xylogalacturonan, arabinan and rhamnogalacturonan II) [71], are targets for pathogens to degrade for nutrients and/or to overcome the physical barriers presented by their host. CWDE can include polygalacturonases, pectate lyases, and pectinesterases which promote pectin degradation [72–78], glucanases (also known as cellulase) which breaks links between glucose residues [79] and xylanases which cleave links in the xylosyl backbone of xyloglucan [80–82].

*Elsinoë fawcettii* effectors and/or CWDE which interact with certain host plant cell wall components could explain the observed host specificity of pathotypes. Computational prediction of genes coding for such virulence factors can lead to many candidate effectors (CE) and potential CWDE, leading to an overabundance of candidates which require prioritisation. This study aimed to generate an assembly of the *E*. *fawcettii* isolate, BRIP 53147a, through whole genome shotgun (WGS) sequencing, to identify candidate virulence genes and appropriately shortlist these predictions to improve the focus of future experimental validation procedures. Computational methods involving genomic, proteomic and comparative analyses enabled the prediction and prioritisation of CE and CWDE which may be interacting with the host plant and overcoming immune defences prior to the biosynthesis of elsinochrome. Additional genes potentially involved in the elsinochrome gene cluster were also predicted, as were additional SM clusters which may be impacting virulence of *E*. *fawcettii*.

## Materials and methods

### Sequencing, assembly, gene prediction, annotation and genomic analyses

*Elsinoë fawcettii* (BRIP 53147a), collected from *C*. *limon* in Montville, Queensland, Australia, was obtained from DAF Biological Collections [11]. The isolate was cultured on potato dextrose agar (Difco) and incubated at 23 to 25°C for two months. Whole genomic DNA was extracted using the DNeasy Plant Mini kit (QIAGEN) according to the manufacturer’s protocol. Paired-end libraries, with a mean insert size of approximately 330 bp, were prepared according to Illumina Nextera^TM^ DNA Flex Library Prep Reference Guide using a Nextera^TM^ DNA Flex Library Prep Kit and Nextera^TM^ DNA CD Indexes. WGS sequencing was performed on Illumina MiSeq platform (600-cycles) at the molecular laboratories of the Centre for Crop Health, USQ. Assembly was performed on the Galaxy-Melbourne/GVL 4.0.0 webserver [83]. Raw reads were quality checked using FastQC (v0.11.5) [84] and trimmed using Trimmomatic (v0.36) [85] with the following parameters: TruSeq3 adapter sequences were removed using default settings, reads were cropped to remove 20 bases from the leading end and 65 bases from the trailing end of each read, minimum quality of leading and trailing bases was set to 30, a sliding window of four bases was used to retain those with an average quality of 30 and the minimum length read retained was 31 bases. *De novo* assembly was performed in two steps, first using Velvet (v1.2.10) [86] and VelvetOptimiser (v2.2.5) [87] with input k-mer size range of 81–101 (step size of 2). Secondly, SPAdes (v3.11.1) [88] was run on trimmed reads with the following parameters: read error correction, careful correction, automatic k-mer values, automatic coverage cutoff and Velvet contigs (>500 bp in length), from the previous step, included as trusted contigs. Contigs >500 bp in length were retained. Reads were mapped back to the assembly using Bowtie2 (v2.2.4) [89] and Picard toolkit (v2.7.1) [90] and visualised using IGV (v2.3.92) [91]. The estimated genome size was determined using Kmergenie (v1.6715) [92] on Galaxy-Australia (v19.09) [93] and GenomeScope [94]. The genome assembly was checked for completeness with BUSCO (v2.0) [95] using the Dothideomycetes orthoDB (v10) dataset [96]. The extent and location of AT-rich regions was determined using OcculterCut (v1.1) [97] with default parameters and mitochondrial contigs removed.

The prediction of genes and transposable elements (TE) was performed on the GenSAS (v6.0) web platform [98], using GeneMarkES (v4.33) [99], with fungal mode, for gene prediction and RepeatMasker (v4.0.7) [100], using the NCBI search engine and slow speed sensitivity, for the prediction of TE. Predicted gene models containing short exons, missing a start or stop codon or which overlapped a TE region were removed from the predicted proteome. The genome was searched for Simple Sequence Repeats (SSR) using the Microsatellite Identification tool (MISA) [101], with the SSR motif minimum length parameters being 10 for mono, 6 for di, and 5 for tri, tetra, penta and hexa motifs.

Annotation was performed using BLASTP (v2.7.1+) [102] to query the *E*. *fawcettii* predicted proteome against the Swiss-Prot Ascomycota database (release 2018_08) [103] with an e-value of 1e-06 and word size of 3. BLAST results were loaded into Blast2GO Basic (v5.2.1) [104], with InterProScan, mapping and annotation steps being performed with default parameters, except HSP-hit coverage cutoff was set to 50% to increase stringency during annotation. Further annotation was achieved using HmmScan in HMMER (v3.2.1) [105] to query the predicted proteome against the Protein Family Database (Pfam) (release 32) [106]. GC% content of the coding DNA sequence (CDS) of each gene was determined using nucBed from Bedtools (v2.27.1) [107]. Predicted proteins were searched for polyamino acid (polyAA) repeats of at least five consecutive amino acid residues using the FIMO motif search tool [108] within the Meme suite (v5.0.2) [109]. The Whole Genome Shotgun project was deposited at DDBJ/ENA/GenBank under the accession SDJM00000000. The version described in this paper is version SDJM00000000. Raw reads were deposited under the SRA accession PRJNA496356.

### Phylogenetic analysis

**Fig 1:**
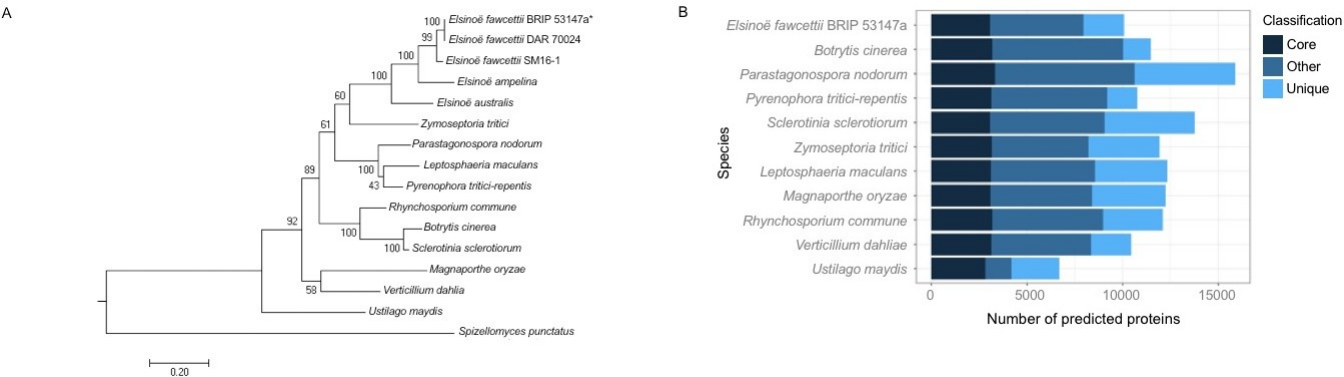
Species comparison. (A) Maximum likelihood phylogenetic tree of *E*. *fawcettii* BRIP 53147a with recently sequenced *Elsinoë* isolates and species included in the comparative study. The phylogenetic tree was inferred from a concatenated dataset including partial TEF1α and RPB2 regions. *Spizellomyces punctatus* was used as the outgroup. The branch length indicates the number of nucleotide substitutions per site, bootstrap values are shown at nodes and the isolate analysed in this study is denoted with asterisk (*). (B) Comparison of gene classifications among the proteomes of 11 fungal pathogens. Genes were categorised using orthoMCL group IDs, or proteinortho if no group was assigned. Genes were considered; (I) core if they were shared by all 11 species; (II) “other” if they were shared by at least two species, but not all; (III) unique if they were found in only one of the 11 species.

**Fig 2:**
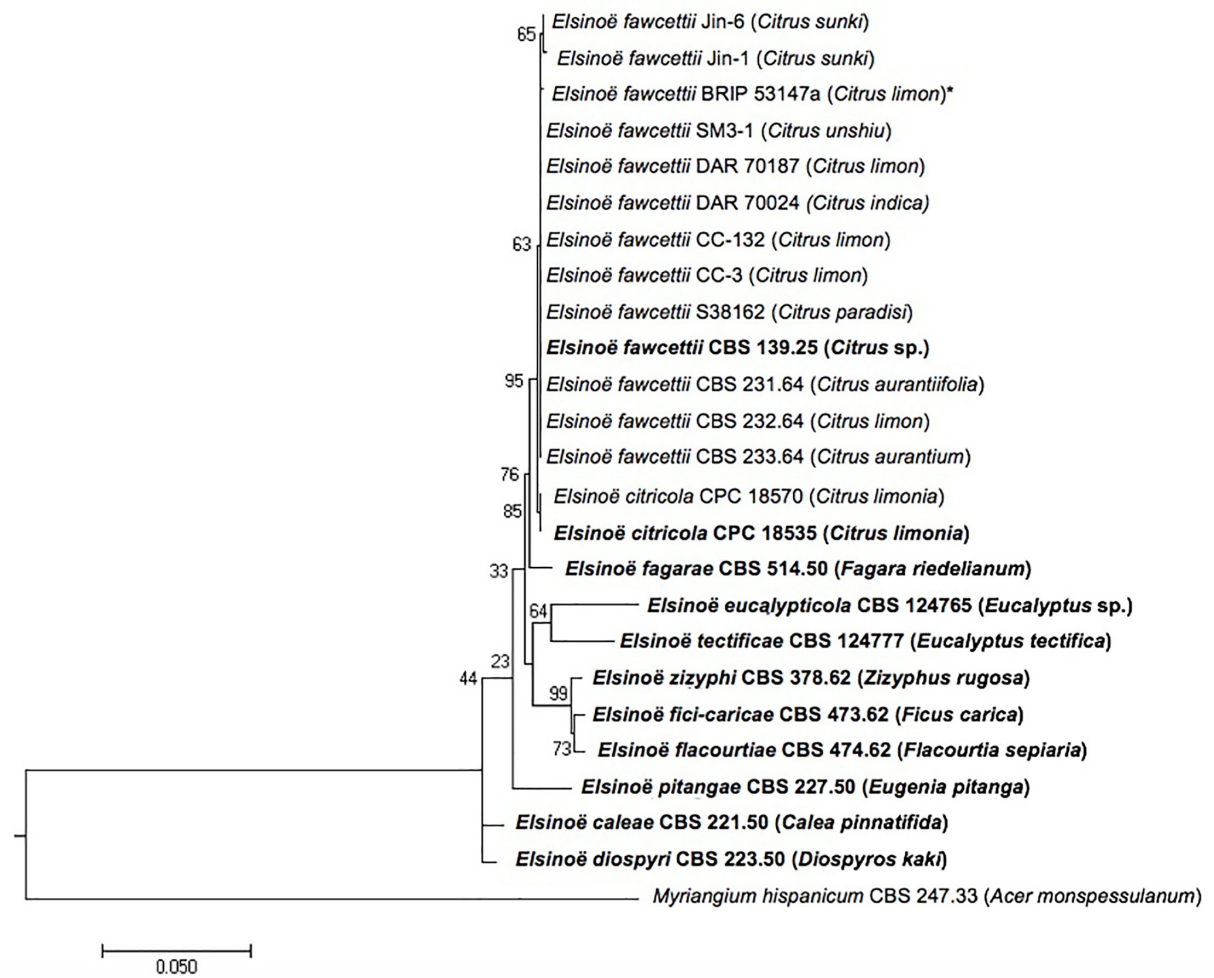
Maximum likelihood phylogenetic tree of *E*. *fawcettii* isolates and closely related species. The phylogenetic tree was inferred from a concatenated dataset including ITS and partial TEF1-α regions. *Myriangium hispanicum* was used as the outgroup. The branch length indicates the number of nucleotide substitutions per site, bootstrap values are shown at nodes, host in parentheses, new isolate described in the current study denoted with asterisk (*) and type strains are in bold.

Two analyses were conducted, the first included three isolates of *Elsinoë fawcettii* (BRIP 53147a, DAR 70024 and SM16-1), and individual isolates of *E*. *ampelina*, *E*. *australis*, *U*. *maydis*, *L*. *maculans*, *M*. *oryzae*, *R*. *commune*, *V*. *dahliae*, *B*. *cinerea*, *Parastagonospora nodorum*, *Pyrenophora tritici-repentis*, *S*. *sclerotiorum* and *Z*. *tritici* and utilised partial TEF1-α and RPB2 regions which were obtained using BLASTN (v2.7.1+) [102] on each assembly; *Spizellomyces punctatus* was included as the outgroup. The second used ITS and partial TEF1-α sequences, obtained from GenBank, of 12 *E*. *fawcettii* pathotypes, 11 closely related *Elsinoë* species, and *Myriangium hispanicum* as the outgroup, for phylogenetic analysis with *E*. *fawcettii* (BRIP 53147a). Genome locations and GenBank accessions of all sequences are provided in S1 Table. Sequences for each locus were aligned using MUSCLE [110] with a gap open penalty of -400, concatenated and used to perform maximum likelihood analysis in MEGA7 [111] based on the General Time Reversible model [112] with partial deletion of 90% and 1000 bootstrap replicates. The initial tree for each maximum likelihood analysis was automatically selected using Neighbor-Join and BioNJ on the matrix of pairwise distances estimated using the Maximum Composite Likelihood method. A discrete Gamma distribution utilising 4 categories (+G, parameter = 0.5348 (Fig 1) and 0.4095 (Fig 2)) was used and the rate variation model allowed some sites to be invariable (+*I*, 15.4278% sites (Fig 1) and 26.6862% sites (Fig 2)). The character matrix and tree were combined and converted to nexus format using Mesquite (v3.6) [113] prior to TreeBASE submission (Fig 1 TreeBASE reviewer access: http://purl.org/phylo/treebase/phylows/study/TB2:S26086?x-access-code=26e5270bba657f28e8d78a0849503953&format=html

Fig 2 TreeBASE reviewer access: http://purl.org/phylo/treebase/phylows/study/TB2:S26087?x-access-code=8130d199a2304fe8bd684df1cc2ebacc&format=html). *E*. *fawcettii* (BRIP 53147a) ITS and partial TEF1-α sequences (accessions MN784182 and MN787508) were submitted to GenBank.

### Sequence information

Genome assemblies and predicted proteomes included in the comparative analysis were obtained from GenBank. These included *U*. *maydis* (accession GCF_000328475.2, no. of scaffolds = 27) [114], *L*. *maculans* (accession GCF_000230375.1, no. of scaffolds = 76) [115], *M*. *oryzae* (accession GCF_000002495.2, no. of scaffolds = 53) [116], *R*. *commune* (accession GCA_900074885.1, no. of scaffolds = 164) [117], *V*. *dahliae* (accession GCF_000150675.1, no. of scaffolds = 55) [118], *B*. *cinerea* (accession GCF_000143535.2, no. of scaffolds = 18) [119], *Parastagonospora nodorum* (accession GCF_000146915.1, no. of scaffolds = 108) [120], *Pyrenophora tritici-repentis* (accession GCA_003231415.1, no. of scaffolds = 3964) [121], *Sclerotinia sclerotiorum* (accession GCF_000146945.2, no. of scaffolds = 37) [122] and *Z*. *tritici* (accession GCA_900184115.1, no. of scaffolds = 20) [123]. Additionally, genome assemblies of *E*. *fawcettii* DAR 70024 (accession GCA_007556565.1, no. of scaffolds = 53), *E*. *fawcettii* SM16-1 (accession GCA_007556535.1, no. of scaffolds = 1,266), *E*. *australis* Ea1 (accession GCA_007556505.1, no. of scaffolds = 21) [124] and *E*. *ampelina* (accession GCA_005959805.1, no. of scaffolds = 13) [125], were obtained from GenBank and gene prediction performed as for *E*. *fawcettii* BRIP 53147a. TE were identified in each assembly, as previously described, and predicted genes which overlapped them were similarly removed from predicted proteomes. Sequences of experimentally verified effector proteins were obtained from the EffectorP 2.0 study [126].

### Prediction of secretome and effectors

Secretome and effector prediction was performed on the predicted proteomes of *E*. *fawcettii* and 10 fungal species known to contain effector proteins. Secretome prediction for each species began with a set of proteins predicted as secreted by either SignalP (v4.1) [127], Phobius [128] or ProtComp-AN (v6) [129]. This set was run through both the TMHMM Server (v2.0) [130] and PredGPI [131] to predict proteins with transmembrane helices and GPI-anchors, respectively. Those proteins with >1 helix or with 1 helix beyond the first 60 amino acids were removed, as were those with “highly probable” or “probable” GPI anchors. Remaining proteins formed the predicted secretome and were subjected to candidate effector prediction using EffectorP (v1.0 and v2.0) [126,132].

### Genomic, proteomic and known effector analyses

Sequences of 42 experimentally verified effector proteins, which showed >98% similarity to proteins from the 10 species included in this study, and which appeared in both the predicted secretome and candidate effector list for the respective species, were utilised in the known effector analysis. The following analyses were performed on the proteome/genome of each species. Results relating to the 42 known effectors were compared to results of all proteins from each species. Length of the intergenic flanking region (IFR) was determined as the number of bases between the CDS of two adjacent genes. Genes were labelled as gene-dense if the IFR on each side was less than 1500 bp, genes on a contig end were not included among gene-dense labelled genes. Genes with IFR greater than or equal to 1500 bp were labelled as gene-sparse genes. SM clusters were predicted by passing genome assemblies and annotation files through antiSMASH fungal (v4.2.0) [133] using the Known Cluster Blast setting. Core, unique and other genes for each species were determined by grouping proteins into ortholog groups using the orthoMCL algorithm [134] followed by ProteinOrtho (v5.16b/v6.0.14) [135] on remaining unclassified genes. For the purposes of CE prioritisation, each *Elsinoë* proteome was compared individually to the proteomes of the 10 fungal species included in the study during ortholog classification. Core genes were those shared by all species included in the comparison, unique genes were found in only one species and other genes were those shared by at least two species, but not all. Additionally, ortholog classification, using the method described above, was performed on the five species of *Elsinoë* together, to determine potential *E*. *fawcettii*- and isolate-specific genes. GC% content of the CDS of each gene was determined as described above, Q_1_ and Q_3_ values were determined for each species using R [136]. HmmScan [105] of all protein sequences against the Pfam database [106] was performed as described above. Genomic AT-rich region identification was performed using OcculterCut (v1.1) [97] as described above. For genomes with identified AT-rich regions, the distance between genes and their closest AT-rich region edge was determined using Bedtools closestBed [107], as was the distance between genes and the closest TE.

### Prioritisation of candidate effectors

CE of each species were prioritised using an optimised scoring system based on the analysis of known effectors in 10 fungal species. All were scored out of at least four points, corresponding to one point allocated for each of the following conditions: (I) not labelled as gene-dense; (II) no involvement in predicted SM clusters; (III) labelled as either unique to the species or allocated to the same orthoMCL group as a known effector; and (IV) GC% of CDS was either below the Q_1_ value or above the Q_3_ value of the respective species. For species with genomes which had >2% TE coverage or >25% AT-rich region coverage, CE were scored out of five points. Those genomes which had both >2% TE and >25% AT-rich region coverage, CE were scored out of six points. Hence, all candidate effectors were scored out of *n* (four, five or six) points, those CE which obtained a score of *n* or *n*-1 points were labelled as prioritised CE. P-values, to test overrepresentation of SP and CE among *E*. *fawcettii*, were determined using the one-tailed Fisher's exact test in R [136].

### Prediction of other virulence genes

SM clusters were predicted using antiSMASH fungal (v4.2.0) [133] as described above. CAZymes were predicted by passing the predicted proteomes through the dbCAN2 meta server [137] and selecting three tools including HMMER scan against the dbCAN HMM database [138], Diamond [139] search against the Carbohydrate-Active enZYmes (CAZy) database [140] and Hotpep query against the Peptide Pattern Recognition library [141]. Predicted CAZymes were taken as those with positive results for at least two out of the three tools. Potential pathogenesis-related proteins were identified by querying the predicted proteomes against the Pathogen Host Interactions Database (PHI-base) (v4.6, release Oct 2018) [142] using BlastP (v2.7.1) [102] analyses with an e-value of 1e-06 and a query coverage hsp of 70%, those results with >40% similarity were retained. Prioritised candidate CWDE were shortlisted from the predicted CAZymes to those which were predicted as secreted and obtained hits to plant associated fungal pathogenicity-related genes in PHI-base which showed evidence of reduced virulence in knockout or mutant experiments.

## Results and discussion

### Genome assembly and features

The genome assembly of *E*. *fawcettii* (BRIP 53147a), deposited at DDBJ/ENA/GenBank (accession SDJM00000000), was sequenced using paired-end Illumina WGS sequencing technology. Assembly of reads produced a draft genome 26.01 Mb in size with a coverage of 193x (Table 1) and consisted of 286 contigs greater than 500 bp in length, with an N50 of 662,293 bp, a mean contig length of 90,948 bp and an overall GC content of 52.3%. The estimated genome size based on k-mer counts of trimmed reads was 27.25–27.28 Mb, indicating approximately 4.7% of the genome may be missing from the current assembly. Running the assembly against the Dothideomycetes orthoDB (v10) [96] showed 94.2% of complete single copy genes were found in the *E*. *fawcettii* assembly, indicating a high degree of coding DNA sequence completeness. The genome of *E*. *fawcettii* is comparable in size to other fungal genomes, including *Eurotium rubrum* (26.21 Mb) [143], *Xylona heveae* (24.34 Mb) [144] and *Acidomyces richmondensis* (29.3 Mb) [145], however it is smaller than the average Ascomycota genome size of 36.91 Mb [146]. When analysed against the 10 fungal species included in this comparative analysis (*B*. *cinerea*, *L*. *maculans*, *M*. *oryzae*, *Parastagonospora nodorum*, *Pyrenophora tritici-repentis*, *R*. *commune*, *V*. *dahliae*, *S*. *sclerotiorum*, *U*. *maydis* and *Z*. *tritici*), the *E*. *fawcettii* assembly is the second smallest, after the *U*. *maydis* assembly at 19.6 Mb. It is comparable in size to two *E*. *fawcettii* genomes recently published, being 26.65 Mb (SM16-1) and 26.32 Mb (DAR 70024) [124]. TE identification, by analysis against Repbase (release 18.02) [147], showed a coverage of only 0.37%, indicating a low proportion of the *E*. *fawcettii* genome is represented by currently known TE, this is a likely contributor to its comparatively small genome size. This low TE coverage may also be the result of a fragmented genome [148]. It is possible, should long read sequencing of this isolate be completed in the future, TE coverage may appear higher.

**Table 1:** Features of *Elsinoë fawcettii* (BRIP 53147a) genome assembly.

General Features	
Assembly length (bp)	26,011,141
Coverage	193x
Number of contigs	286
Mean GC content (%)	52.3
N50 (bp)	694,004
Mean contig length (bp)	90,948
Minimum contig length (bp)	501
Maximum contig length (bp)	2,345,732
Coverage of interspersed repeats (bp)	95,654 (0.37%)
Coverage of short simple repeats (bp)	6868 (0.026%)
Number of predicted gene models	10,080
Number of contigs containing predicted genes	141
Mean gene length (bp)	1,573
Mean number of exons per gene	2.35
Number of genes containing a polyAA repeat	1,073
Mean GC content of CDS (%)	54.7

The *E*. *fawcettii* genome has less predicted gene models than the average Ascomycota genome of 11129.45 [146]. Gene prediction produced 10,080 gene models, which is comparable to the number of genes predicted for the recently published *E*. *fawcettii* genomes, specifically 10,340 (SM16-1) and 9930 (DAR 70024) genes [124]. A total of 5,636 (55.91%) genes were annotated, while 4,444 (44.09%) were labelled as coding for hypothetical proteins. The average gene length was 1,573 bp with an average of 2.35 exons per gene, there were 3,280 single exon genes. The mean GC content of CDS was 54.7%, which was 2.4% higher than the overall GC content and showed a wide variation in range, with the lowest scoring gene at 44.29% GC and the highest being 71.53%, thus exposing a spectrum on which genes may be differentiated. Hmmscan [105] analysis of the predicted proteome against the Pfam database [106] revealed a high proportion (70.1% = 7,069) of genes with at least one hit to a Pfam model. The same analysis performed on the proteomes of the 10 fungal species included in the comparative analysis gave results ranging from 48.6% for *S*. *sclerotiorum*, with the lowest proportion of Pfam hits, to 74.9% for *U*. *maydis* with the highest, and a mean of 62.1% over the 11 species (S2 Table).

Phylogenetic analysis showed all species included in this comparative study are distinct from one another, with only the three *E*. *fawcettii* isolates being closely related (Fig 1A). Analysis of orthologous genes among *E*. *fawcettii* and the 10 comparative species (other *Elsinoë* species not included) indicated 3,077 (30.5%) of the predicted genes of *E*. *fawcettii* were core genes, finding hits through OrthoMCL or ProteinOrtho in all 11 species (S2 Table). There were 4,874 (48.4%) *E*. *fawcettii* genes found in at least one other species but not all and were therefore labelled as “other” genes. Lastly, the remaining 2,129 (21.1%) were found in only the *E*. *fawcettii* proteome, 140 of these, however, obtained a hit to an orthoMCL group and were therefore set aside and not considered as unique proteins in subsequent analyses, leaving 1,989 (19.7%) genes presumed to be *Elsinoë*-specific and therefore potentially involved in either *Elsinoë*- or *E*. *fawcettii*-specific pathogenesis pathways. The comparative analysis among the core, unique and other genes of the 11 species (S2 Table) (Fig 1B) indicated that *U*. *maydis* was set apart from the other species by showing the lowest proportion of “other” genes and the highest proportion of unique genes, this was expected as *U*. *maydis* was the only biotroph and Basidiomycete among the group, and is seen separated from the Ascomycota clade in the phylogenetic analysis (Fig 1A). E. *fawcettii* showed a below average percentage of unique genes which may be expected due its smaller than average sized genome and proteome. When comparing predicted genes of *E*. *fawcettii* (BRIP 53147a) to those of *E*. *ampelina*, *E*. *australis* and two *E*. *fawcettii* isolates (Table 2), 75.70% (7,631) of genes were labelled as core genes, indicating the majority of genes appeared in all five isolates. A further 12.37% (1,247) were classed as accessory, being found in more than one species, but not all five isolates. *E*. *fawcettii*-specific genes, found in at least two *E*. *fawcettii* isolates, accounted for 10.72% (1081), while *E*. *fawcettii* BRIP 53147a-specific genes made up 1.2% (121) of genes. The results of Table 2 indicated that the predicted gene repertoire of *E*. *fawcettii* isolates BRIP 53147a and DAR 70024 were closely aligned, with isolate SM16-1 set apart with a higher proportion of unique genes. As SM16-1 is classified as the FBHR pathotype and DAR 70024 as the Tyron’s pathotype, SM16-1 therefore has the ability to infect a wider variety of host plants [3], these additional unique genes of *E*. *fawcettii* SM16-1 may contribute to its greater host range.

**Table 2:** Summary gene classifications among five *Elsinoë* species.

Classification	*E*. *fawcettii* BRIP 53147a	*E*. *fawcettii* DAR 70024	*E*. *fawcettii* SM16-1		*E*. *australis* Ea-1	*E*. *ampelina* YL-1
Core	75.70%	74.79%	73.08%	80.35%		75.21%
Accessory	12.37%	12.96%	12.34%	9.67%		12.58%
*E*. *fawcettii*-specific	10.72%	10.84%	8.16%	N/A		N/A
Unique	1.20%	1.26%	6.43%	9.98%		12.21%

The overall GC content of *E*. *fawcettii* was 52.3%. However, when taking AT-rich regions into consideration, 98.97% of the genome had an average GC content of 52.8%, while the remaining 1.03% consisted of AT-rich regions with an average GC content of 33.8%. AT-rich regions are sections of DNA that are scattered throughout the genome and have a significantly higher AT content compared to adjacent GC equilibrated blocks [97]. The presence of AT-rich regions in genomes varies widely, for example *Sclerotinia sclerotiorum* does not show evidence of AT-rich regions [149], while 36% of the *L*. *maculans* genome is covered by AT-rich regions which have an average GC content of 33.9% [47]. AT-rich regions are thought to develop in, and nearby to, regions containing TE repeats, through Repeat-Induced Point mutation (RIP), a mechanism used to inhibit the destructive actions of TE against an organism’s genome. Through a fungal genome defence mechanism causing cytosine to thymine polymorphisms, a TE repeat sequence is inhibited from further movement and potential destruction of necessary genes. This same type of polymorphism can also occur in genes nearby to TE regions [150–153], potentially providing numerous genomic locations with increased plasticity scattered throughout the genome. While RIP occurs during the sexual phase it has also been observed in asexual fungi and is thought to indicate a species reproductive history or potential [154]. AT-rich regions are present within the *E*. *fawcettii* genome, however the extent of their coverage in the present assembly is low, 59 regions with an average GC content of 33.8% cover only 1.03% of the genome. Sixteen regions are found overlapping TE, while four are found within 2 Kb of a TE region, meaning 33.9% of the AT-rich regions potentially represent RIP-affected regions. The remaining 66.1%, found either >2Kb away or on a contig that does not contain a predicted TE region, are potentially RIP-affected regions where the TE is no longer recognisable. The AT-rich regions of *E*. *fawcettii* are not scattered evenly throughout the genome, instead 29/59 (49.2%) are situated at the end of a contig and 15/59 (25.4%) cover the entire length of a contig, specifically contigs not containing genes. Two further AT-rich regions were located between the end of a contig and the beginning of the first gene and so were grouped with those located at the end of a contig. The remaining 13 regions (22.0%) were situated within a contig with genes residing on both sides. Hence, the majority either made up the end of a contig which contained genes or filled entire contigs which did not contain genes, meaning it is likely that the sequence of many *E*. *fawcettii* AT-rich regions contain sections of such low complexity that contig breaks result, a hypothesis which could be tested in the future using long read sequencing technology. Eight predicted genes at least partially overlap these regions and 57 are located within 2 Kb, a finding which has potential significance as AT-rich regions have been known to harbour effector genes in fungal pathogens [155, 156]. There was a large range of diversity of AT-rich region coverage among the fungal pathogens analysed in the current study; *S*. *sclerotiorum*, *Pyrenophora tritici-repentis*, *M*. *oryzae* and *U*. *maydis* showed no AT-rich regions; *V*. *dahliae* (1.5%), *B*. *cinerea* (4.9%), *Parastagonospora nodorum* (6.6%) and *Z*. *tritici* (17.3%) showed lower degrees of AT-rich coverage; while *R*. *commune* (29.5%) and *L*. *maculans* (37%) showed the greatest extent. These levels of AT-rich coverage did not appear to correlate with pathogen classification as necrotrophic, hemibiotrophic or biotrophic, nor as host-specific or broad-host range pathogens. The genomic location of AT-rich regions was, however, further included in the known effectors and candidate effectors analyses.

Identification and analysis of SSR in the *E*. *fawcettii* genome located 400 regions covering 6,868 bp (0.026%), 164 (41%) of which were contained within a predicted gene. Furthermore, polyAA repeats, of at least five identical and adjacent residues, were identified within 1,073 predicted protein sequences. The presence of repetitive sequences has been noted in fungal effectors [33, 45, 157] and implicated in the function and evolution of pathogenicity-related genes of other plant-associated microorganisms [158]. Hence, SSR- and polyAA-containing proteins were retained for cross-referencing against candidate effectors.

Phylogenetic analysis of partial ITS and TEF1-α regions of *E*. *fawcettii* (BRIP 53147a) in comparison with other *E*. *fawcettii* isolates and closely related *Elsinoë* species (Fig 2) indicates *E*. *fawcettii* (BRIP 53147a) closely aligns with the *E*. *fawcettii* clade. Substitutions appearing in the Jingeul pathotype isolates are not seen in isolate BRIP 53147a. One G to A substitution in the TEF1-α region sets isolate BRIP 53147a apart from the other *E*. *fawcettii* isolates (S3 Table), a base which is at the 3^rd^ position of a Glu codon and hence does not result in a translational difference. This substitution in the BRIP 52147a isolate appeared with a high degree of confidence, 100% of sequence reads aligned back to the assembly and a coverage of 241x, at this point, agreed with the substitution. While it is thought that isolate BRIP 53147a belongs to either the Lemon or Tyron’s pathotype, the only two pathotypes reported in Australia [2, 3, 7], it is yet to be determined which or if it constitutes a new pathotype of its own. Aside from the one base substitution in the TEF1-α region, there would be some expected differences throughout the genomes of the *E*. *fawcettii* BRIP 53147a isolate and the other *E*. *fawcettii* isolates due to differences in collection details, such as geographical location, year and host specificity. Specifically, isolate BRIP 53147a was collected in Montville, Queensland in 2009, while the other Australian isolates, DAR 70187 and DAR 70024, belonging to the Lemon and Tyron’s pathotypes, were collected 15 years earlier in Somersby and Narara in NSW, respectively [7], both a distance of almost 1000 km away. Several isolates from Fig 2 have been tested for host pathogenicity leading to the designation of specific pathotypes [3], as opposed to relying on only sequence data and thus illustrating the importance of experimental validation prior to pathotype or species classification. For example, Jin-1 and Jin-6 are classified as the Jingeul pathotype, SM3-1 as FBHR, S38162 as FNHR, CC-132 as SRGC, DAR 70187 and CC-3 as the Lemon pathotype, and DAR 70024 as Tyron’s pathotype [3]. Host specificity experimentation for the *E*. *fawcettii* BRIP 53147a isolate is a suggested future step, as is the whole genome sequencing and analysis of further *E*. *fawcettii* isolates for comparison. The comprehensive host pathogenicity testing of 61 *E*. *fawcettii* isolates and their subsequent classification into six pathotypes [3] coupled with genomic sequencing data analysis would provide a wealth of knowledge of potential host-specific pathogenicity-related genes and mutations.

### Prediction of secretome and effectors

**Fig 3:**
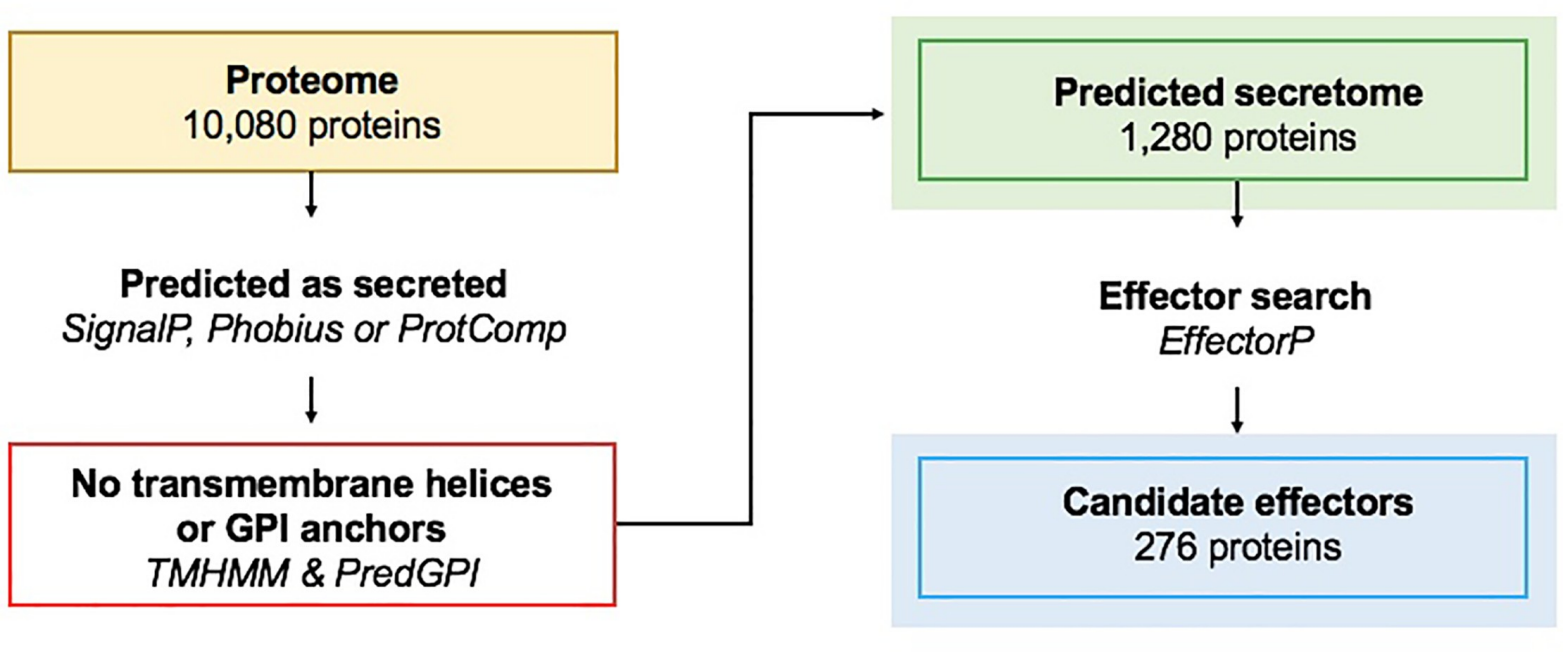
Pipeline for the discovery of the predicted secretome and candidate effectors. The secretome search started with the predicted proteins of a species, proteins were predicted as secreted using at least one of three tools, proteins with predicted transmembrane helices or GPI-anchors were removed. Candidate effectors were predicted using EffectorP (v1.0 and v2.0). The number of proteins shown for the predicted proteome, secretome and effectome refers to the *Elsinoë fawcettii* BRIP 53147a genome.

A total of 1,280 genes (12.7% of the proteome) were predicted to code for secreted proteins (SP) in the *E*. *fawcettii* (BRIP 53147a) genome (Table 3). Using the discovery pipeline outlined in Fig 3, classically secreted proteins with a detectable signal peptide were predicted by either SignalP and/or Phobius providing 1,449 proteins, while ProtComp identified a further 120 as potential non-classically secreted proteins. Of these 1,569 proteins, 186 were removed as they were predicted to contain transmembrane helices, an indication that while targeted for secretion, the protein likely functions while situated in the cell membrane. A further 103 were removed as they contained a predicted GPI anchor, also suggesting they associate with the cell membrane to perform their function, leaving a total of 1,280 proteins identified as likely SP. To enable comparison of the species’ predicted secretomes and CE, the same prediction pipeline (Fig 3) was used on the proteomes of 14 further fungal species included in the analysis (Table 3), essentially utilising additional *Elsinoë* genomes and genomes which contain known protein effectors for comparison. The proportion of predicted SP in the *E*. *fawcettii* proteome was similar to that of other necrotrophic fungal pathogens, which ranged from *B*. *cinerea* at 11.3% to *Parastagonospora nodorum* at 13.9%.

**Table 3:** Predicted Secreted Proteins (SP), Candidate Effectors (CE) and known effectors.

Species	Total proteins*	SP (% of total)	CE (% of SP)	Known effectors correctly predicted as SP and CE	Known effectors not predicted as SP and CE
**Necrotrophs:**					
***Elsinoë fawcettii* BRIP 53147a**	10,080	1,280 (12.7%)	276 (21.6%)	-	
***Elsinoë fawcettii* DAR 70024**	10,223	1,291	274	-	
***Elsinoë fawcettii* SM16-1**	10,519	1,393	317	-	
***Elsinoë australis***	9,253	1,091	235	-	
***Elsinoë ampelina***	9,804	1,167	270	-	
***Botrytis cinerea***	11,481	1,294 (11.3%)	285 (22.0%)	NEP1	
***Parastagonospora nodorum***	15,878	2,206 (13.9%)	932 (42.2%)	Tox1, ToxA	
***Pyrenophora tritici-repentis***	10,771	1,298 (12.1%)	388 (29.9%)	ToxB	
***Sclerotinia sclerotiorum***	13,770	1,707 (12.4%)	692 (40.5%)	SsSSVP1	
***Zymoseptoria tritici***	11,936	1,514 (12.7%)	597 (39.4%)	Zt6, AvrStb6	
**Hemibiotrophs:**					
***Leptosphaeria maculans***	12,337	1,883 (15.3%)	787 (41.8%)	AvrLM6, AvrLM11, AvrLM4-7	
***Magnaporthe oryzae***	12,236	2,263 (18.5%)	1055 (46.6%)	SPD10, Msp1, BAS1, SPD4, SPD2, MoCDIP3, MoCDIP4, AVR-Pik, MoCDIP1, Bas107, BAS2, BAS3, BAS4, Avr-Pita1, Bas162, MoHEG13, SPD7, MC69, AvrPi9, AvrPiz-t, SPD9, MoCDIP5	MoCDIP2
***Rhynchosporium commune***	12,100	1,510 (12.5%)	509 (33.7%)	NIP1, NIP2, NIP3	
***Verticillium dahliae***	10,441	1,407 (13.5%)	413 (29.4%)	PevD1, VdSCP7	Vdlsc1
**Biotroph:**					
***Ustilago maydis***	6,692	856 (12.8%)	256 (29.9%)	Pit2, Pep1, See1, Cmu1, Tin2	Eff1-1

*Not including gene models which overlap a predicted TE region

Known effectors were frequently identified by the CE pipeline (Fig 3), with 43/45 (95.6%) correctly predicted as being secreted and 42/45 (93.3%) also predicted as effectors (Table 3). This high proportion of predicted effectors is due to 23 being utilised as positive training data for EffectorP (v2), the unbiased sensitivity and specificity of EffectorP (v2) is reportedly 84.5% and 82.8%, respectively [126]. Those known effectors which were tested but not identified as SP included Vdlsc1 (*V*. *dahliae*) and MoCDIP2 (*M*. *oryzae*). Vdlsc1 lacks an N-terminal signal peptide and is unconventionally secreted [159], however it was not identified as a non-classically secreted protein. MoCDIP2 was removed as it obtained a GPI-anchor hit. Additionally, Eff1-1 (*U*. *maydis*) was predicted as secreted but not as a candidate effector, Eff1-1, along with MoCDIP2, are both known false negatives of EffectorP 2.0 [126].

The total number of CE identified for *E*. *fawcettii* (BRIP 53147a) was 276, meaning only 21.6% of SP gained CE classification, this was the lowest proportion out of all 11 species analysed (Table 3). This may be explained by the potential favouring of EffectorP towards SP of species on which it was trained. To further investigate this potential, results of EffectorP for the 11 species were compared to the results of an alternate candidate effector search; SP with a protein length less than the species’ median and with no Pfam hit other than to that of a known effector (S4 Table). While this second method resulted in the identification of a higher number of CE for each species, *E*. *fawcettii* still obtained the lowest proportion of CE out of predicted SP. It also highlighted the advantage of using EffectorP to narrow down an extensive catalogue of SP, as opposed to identifying CE based on arbitrary features. However, the CE predicted by EffectorP still range in the hundreds (Table 3), it was therefore beneficial to further shortlist candidates for prioritisation. To achieve this, known effectors which were correctly predicted as both SP and as CE (Table 3) were retained for further analysis to generate an optimised prioritisation scoring system. In the current study, positive results from either version of EffectorP (1.0/2.0) [126,132] formed the CE set, while this provided a larger group for subsequent prioritisation, it removed potential user discrimination based on arbitrary features. EffectorP 1.0 has previously been shown to predict effectors with a shorter average sequence length compared to version 2.0 and, additionally, selecting only CEs predicted by both versions’ favours proteins with a higher cysteine content [126]. By utilising a CE set predicted by either version potentially allows greater variety of CE prediction.

### Known effector analysis

A total of 42 known effectors from 10 fungal species were analysed for; (I) gene density; (II) GC content; (III) involvement in SM clusters; (IV) uniqueness; (V) distance to the closest TE; and (VI) distance to the closest AT-rich regions (Table 4). Results were compared to those of all predicted genes from each of the same 10 species (S5 Table). Features observed at a higher rate among the known effector group compared with each species’ proteome were used to prioritise CE using a point allocation system. (I) Genes were labelled as gene-dense if the IFRs on both sides were less than 1500 bp. The proportions of gene-dense genes ranged from 17.66% (*Pyrenophora tritici-repentis*) to 70.23% (*L*. *maculans*) (S5 Table) with a mean of 49.4%, in contrast to 9/42 (21.4%) known effectors (Table 4). This provided grounds to allocate one point to each known effector which was not labelled as gene-dense. (II) GC content of the CDS of each gene was determined and median values calculated for each species, revealing the GC percentage of 32/42 (76.2%) known effectors fell either below the Q_1_ value or above the Q_3_ value for the respective species. When compared to an expected 50% in the upper and lower quartiles, this provided reason for the allocation of one point to known effectors should they fall in these two quartiles. (III) No overlap was observed between known effectors and the predicted SM clusters within each species, giving strong reason for the allocation of one point to known effectors that were not included in SM clusters. (IV) Analysis of gene classification (core, unique or other) for each known effector highlighted that 41/42 (97.6%) were either unique to the species (31/42) or were assigned an orthoMCL group ID of a known effector (10/42). In contrast, the proportion of unique genes for each species was much lower, ranging from 11.9% (*B*. *cinerea*) to 33.7% (*S*. *sclerotiorum*), with an average of 25.4%. The proportion of genes allocated an orthoMCL of a known effector was similarly low at less than 0.3% for all species. Thus, a point was allocated to known effectors that were either unique to the species or obtained the same orthoMCL ID of a known effector. (V) Those genomes with >2% TE coverage also showed a high proportion of known effectors in the close vicinity of TE. Specifically, 29/32 (90.6%) known effectors from *Z*. *tritici*, *S*. *sclerotiorum*, *B*. *cinerea*, *R*. *commune*, *L*. *maculans* and *M*. *oryzae* were within 20 Kb of a TE region, compared to an average of 47.1% of genes within 20 Kb of a TE for the same six species. This led to the allocation of one point for known effectors less than 20 Kb from a TE for species with >2% TE coverage. (VI) Lastly, of the genomes analysed, only those consisting of >25% AT-rich regions, being *R*. *commune* and *L*. *maculans*, were found to have a noticeable association between the location of known effectors and AT-rich regions. The distance of all known effectors to the closest AT-rich region, of these two species, were found to be less than the Q_1_ value for each species. Hence, known effectors with these specifications, in species with >25% AT-rich region coverage, were allocated one point. It can be seen that depending on the degree of TE and AT-rich region coverage, each species’ known effectors may be scored out of four, five or six points, henceforth referred to as “*n* points”. Over the 10 species with known effectors which were analysed, Table 4 illustrates a total of 38/42 (90.5%) known effectors obtained *n* or *n*-1 points, revealing a process which could be used to prioritise the many CE predicted for the *E*. *fawcettii* genome.

**Table 4:** Features of known fungal effectors used to guide candidate effector prioritisation.

Effector	Gene density class	CDS GC%	Within SM gene cluster	Ortholog class	Distance to TE	Distance to AT-rich region	Total possible points (*n* points)	Points scored
**Necrotrophic:**								
***Botrytis cinerea*:**								
NEP1	Sparse	>Q_3_	No	Other^A^	16,435	N/A	5	5
***Parastagonospora nodorum*:**								
Tox1		<Q_1_	No	Unique	N/A	N/A	4	4
ToxA	Sparse	<Q_1_	No	Unique	N/A	N/A	4	4
***Pyrenophora tritici-repentis*:**								
ToxB		<Q_1_	No	Unique	N/A	N/A	4	4
***Sclerotinia sclerotiorum*:**								
SsSSVP1	Sparse	Q_2_^B^	No	Unique	8,521	N/A	5	4
***Zymoseptoria tritici*:**								
Zt6	Dense^B^	>Q_3_	No	Core^A^	14,100	N/A	5	4
AvrStb6	Sparse	>Q_3_	No	Unique	3,166	N/A	5	5
**Hemibiotrophic:**								
***Leptosphaeria maculans*:**								
AvrLM6		<Q_1_	No	Unique	3,766	0	6	6
AvrLM11	Sparse	<Q_1_	No	Unique	2,467	0	6	6
AvrLM4-7	Sparse	<Q_1_	No	Unique	891	0	6	6
***Magnaporthe oryzae*:**								
SPD10	Dense^B^	Q_2_^B^	No	Unique	8,747	N/A	5	3^C^
Msp1	Dense^B^	>Q_3_	No	Other^A^	39,744^B^	N/A	5	3^C^
BAS1	Sparse	<Q_1_	No	Unique	249	N/A	5	5
SPD4		<Q_1_	No	Unique	1,038	N/A	5	5
SPD2	Dense^B^	>Q_3_	No	Unique	17,554	N/A	5	4
MoCDIP3	Sparse	>Q_3_	No	Unique	168	N/A	5	5
MoCDIP4	Sparse	>Q_3_	No	Other^A^	238	N/A	5	5
AVR-Pik	Sparse	<Q_1_	No	Unique	442	N/A	5	5
MoCDIP1	Sparse	>Q_3_	No	Other^A^	68,564^B^	N/A	5	4
Bas107		<Q_1_	No	Unique	7,541	N/A	5	5
BAS2	Dense^B^	Q_2_^B^	No	Other^B^	4,583	N/A	5	2^C^
BAS4	Sparse	Q_2_^B^	No	Unique	3,898	N/A	5	4
BAS3		Q_2_^B^	No	Unique	12,126	N/A	5	4
Avr-Pita1		<Q_1_	No	Other^A^	299	N/A	5	5
Bas162		<Q_1_	No	Unique	8,604	N/A	5	5
MoHEG13		<Q_1_	No	Unique	5,888	N/A	5	5
SPD7		<Q_1_	No	Unique	8,963	N/A	5	5
MC69	Sparse	>Q_3_	No	Other^A^	18,884	N/A	5	5
AvrPi9	Dense^B^	>Q_3_	No	Other^A^	5,031	N/A	5	4
AvrPiz-t		Q_2_^B^	No	Unique	465	N/A	5	4
SPD9		Q_2_^B^	No	Unique	3,433	N/A	5	4
MoCDIP5	Dense^B^	>Q_3_	No	Other^A^	5,123	N/A	5	4
***Rhynchosporium commune*:**								
NIP3		Q_2_^B^	No	Unique	32,352^B^	1,368	6	4^C^
NIP1	Sparse	>Q_3_	No	Unique	2,611	1,814	6	6
NIP2	Sparse	>Q_3_	No	Unique	1,860	6,572	6	6
***Verticillium dahliae*:**								
PevD1		>Q_3_	No	Other^A^	N/A	N/A	4	4
VdSCP7		Q_2_^B^	No	Unique	N/A	N/A	4	3
**Biotrophic:**								
***Ustilago maydis*:**								
Pit2		<Q_1_	No	Unique	N/A	N/A	4	4
Pep1	Sparse	Q_2_^B^	No	Unique	N/A	N/A	4	3
See1	Dense^B^	<Q_1_	No	Unique	N/A	N/A	4	3
Cmu1	Dense^B^	>Q_3_	No	Unique	N/A	N/A	4	3
Tin2		<Q_1_	No	Unique	N/A	N/A	4	4

^A^ Allocated the same orthoMCL group ID as a known effector

^B^ Possible point not allocated

^C^ Less than *n*-1 points scored

### Prioritisation of candidate effectors

CE were prioritised using the method described above for the analysis of known effectors. The three *E*. *fawcettii* isolates, *E*. *ampelina*, *E*. *australis*, *Parastagonospora nodorum*, *Pyrenophora tritici-repentis*, *V*. *dahlia* and *U*. *maydis* each had <2% TE coverage and <25% coverage of AT-rich regions, their CE were therefore scored out of four points. *Z*. *tritici*, *S*. *sclerotiorum*, *B*. *cinerea* and *M*. *oryzae* had >2% TE coverage but <25% coverage of AT-rich regions and so were scored out of five points. Only the assemblies of *R*. *commune* and *L*. *maculans* showed >2% TE and >25% AT-rich regions, and as such their CE were scored out of six points. By using *n* or *n*-1 points as an acceptable score for CE prioritisation, CE of the 15 pathogens could be reduced, by 31.96% - 77.13% (average 54.39%) (S6 Table), with species that were scored out of more points achieving higher reductions.

**Fig 4:**
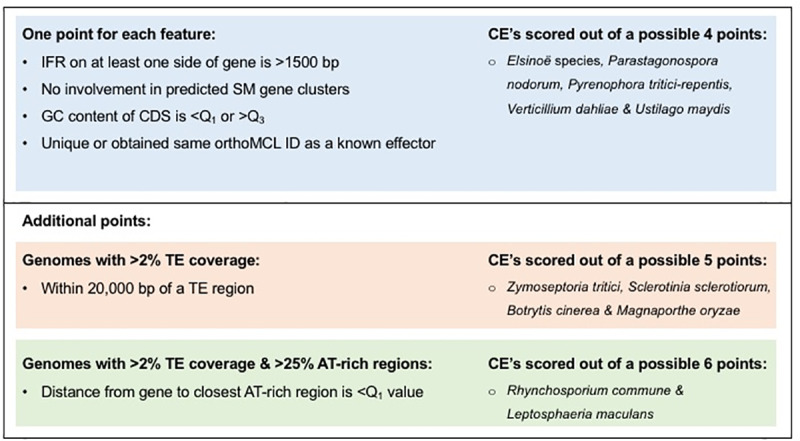
Candidate effector prioritisation features and points. The candidate effectors (CE) of all genomes analysed were scored using features shown in the blue box. Additional features were considered for CE from genomes with >2% TE coverage (red box) and >25% AT-rich region coverage (green box).

**Fig 5:**
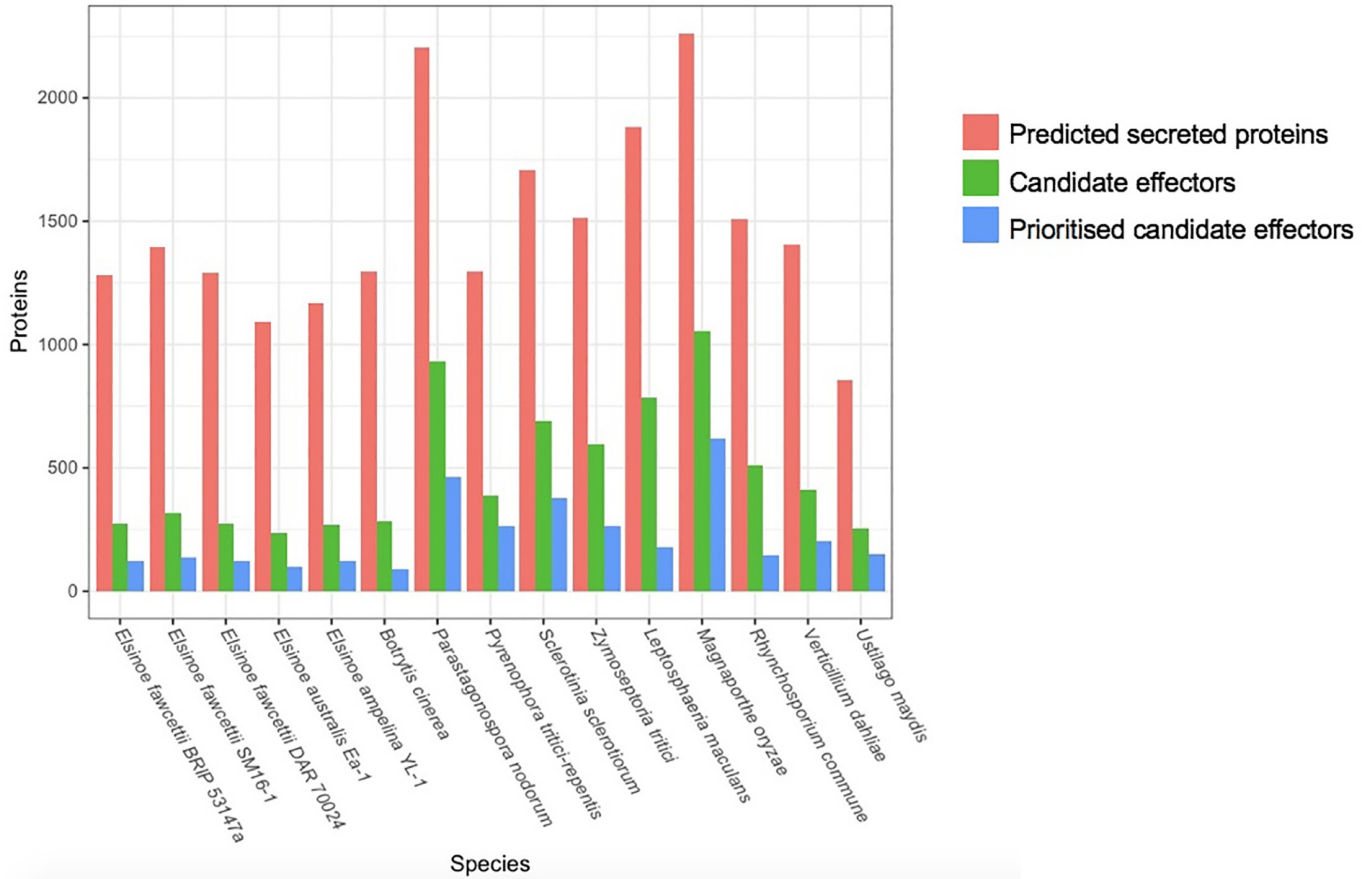
Comparison of numbers of secreted proteins, candidate effectors and prioritised candidate effectors among 15 fungal pathogens. Secreted proteins and candidate effectors were predicted using the pipeline in Fig 3. Prioritised candidate effectors were determined using features shown in Fig 4.

Applying the method outlined in Fig 4 to the CE of *E*. *fawcettii* led to the prioritisation of 120 CE, a reduction of 56.5%, for future experimental validation. This is a comparable reduction to that of the other necrotrophic pathogens (Fig 5, S6 Table), for which six out of seven known effectors were retained within the shortlisted CE. Features of the 120 CE of *E*. *fawcettii* (BRIP 53147a) (S7 Table) indicated many were small in size, had a high GC content, had a high proportion of cysteine residues and were more likely to be classified as gene-sparse. The median protein length was 180 aa, compared to 409 aa for all *E*. *fawcettii* predicted genes. The mean GC content was 57.05% and the mean cysteine content was 2.9%, compared to 54.16% and 1.2%, respectively for all predicted genes of *E*. *fawcettii*. The high proportion (18.3%) of gene-sparse genes among prioritised CE was expected, as CE which were not classified as gene-dense were favoured during the prioritisation process, however high proportions of gene-sparse genes were also observed among the SP and CE (Table 5). Specifically, 4.0% of all *E*. *fawcettii* (BRIP 53147a) predicted genes were classed as gene-sparse, 72.6% as gene-dense and the remaining 23.4% classed as neither. In comparison, 4.8% of SP (*p* = 0.09548) and 8.3% of CE (*p* = 0.00082) were classed as gene-sparse, potentially indicating a preference for gene-sparse locations by CE and proteins likely secreted by the pathogen. PolyAA repeat-containing proteins were not overrepresented among the prioritised CE, only six (5.0%) were found to contain five or more consecutive amino acids, compared to 10.6% of all proteins. Additionally, no CE were found to contain SSR suggesting that diversity of *E*. *fawcettii* effector sequences is not being generated through an increased mutational rate related to short repetitive sequences. Furthermore, the prioritised CE were found scattered throughout the genome over 45 of the 141 gene-containing contigs and did not appear to cluster together. While AT-rich regions were not taken into consideration during the prioritisation of *E*. *fawcettii* CE, due to a low AT-rich coverage of 1.03%, it should be noted that a higher proportion of CE were found among genes on the end of a contig, and significantly more SP and CE were located within 2 Kb of an AT-rich region, than expected. Out of the 252 genes found at the end of a contig 14 (5.6%, *p* = 0.00947) were CE, compared to 2.7% out of all *E*. *fawcettii* proteins. Additionally, of the 57 genes found within 2 Kb of an AT-rich region (including those found to overlap an AT-rich region), 12 (21.1%, *p* = 0.05148) were SP and six (10.5%, *p* = 0.00449) were CE (S7 Table). This suggests that genomic regions near contig breaks, such as sequences of low complexity or regions under-represented by short read sequencing technology, and AT-rich regions may be indicators within the *E*. *fawcettii* genome of nearby effector genes. Interestingly, SP and CE were not overrepresented among genes found within 2 Kb of a predicted TE region, of the 120 genes found in these regions 12 (10%) were SP and 3 (2.5%) were CE, both slightly less than their proportions across the whole genome. This suggested while potential effector genes are more likely to be found near AT-rich regions, a nearby predictable TE region was not necessary. Thus, *E*. *fawcettii*, a necrotrophic pathogen not considered at first thought to utilise protein effectors to increase virulence, shows a subtle, yet intriguing, pattern of CE near AT-rich regions, at contig ends and in more gene-sparse locations. This potentially points towards a set of virulence-related genes being maintained in specific genomic locations and therefore suggesting their potential significance.

**Table 5:** Gene density classification of *Elsinoë fawcettii* (BRIP 53147a) predicted proteins.

Classification	All predicted proteins	Secreted proteins	Candidate effectors	Prioritised candidate effectors
**Gene-sparse**	4.0%	4.8%	8.3%	18.3%
**Gene-dense**	72.6%	68.5%	64.5%	35.0%
**Neither**	23.4%	26.7%	27.2%	46.6%

While analysing proteins using the features mentioned above can shortlist CE, awareness of limitations should be considered. For example, only prioritising CE which are unique to a species, or obtain the same orthoMCL hit as a known effector, limits the identification of novel effectors which may be utilised by multiple species. Hence, a blast search of *E*. *fawcettii* CE against CE of the 10 other fungal pathogens was conducted and indicated 16 (5.8%) *E*. *fawcettii* CE had >70% similarity to at least one candidate effector of another species (S7 Table). Five of these 16 proteins were prioritised CE, one of which had 72.9% similarity to MoCDIP1 (*M*. *oryzae*), a known effector which is expressed in planta and induces host cell death [49], thus highlighting this CE for further investigation. Cross referencing the *E*. *fawcettii* (BRIP 53147a) CE with PHI-base showed 7.2% (20) obtained a hit with >40% similarity, the majority (17/20) of which were core genes found among the *Elsinoë* species studied (S7 Table), indicating a subset of CE which may be of benefit to multiple pathogens.

### Prediction and prioritisation of cell wall degrading enzymes

Further potential pathogenicity-related genes of *E*. *fawcettii* which deserve attention include CWDE. The *E*. *fawcettii* (BRIP 53147a) proteome showed 378 (3.75%) predicted CAZymes (S8 Table), comparable to the proportion of CAZymes seen in the other 10 pathogen genomes, which ranged from 2.8% (*S*. *sclerotiorum*) to 4.3% (*V*. *dahliae*) (S2 Table). Of the total *E*. *fawcettii* CAZymes, 203 (53.7%) were also predicted as secreted, highlighting numerous potential CWDE secreted by the pathogen and targeted for interaction with host carbohydrates. It would be beneficial to compare these potential CWDE with transcriptomic data once available, however, currently they can be cross-referenced against the Pfam database. Analysis of the 203 potential CWDE revealed frequently appearing Pfam hits to pectate lyase and pectinesterase (19 hits), the glycosyl hydrolases family 28 of pectin-degrading polygalacturonases (11 hits) and the glycosyl hydrolases family 43 of hemicellulose-degrading beta-xylosidases (10 hits). Hemicellulose- and pectin-degrading enzymes target plant cell wall components including xyloglucans and pectin’s, respectively [68], both found in high proportions in the primary cell wall, potentially revealing an arsenal of CWDE of *E*. *fawcettii* which are targeted towards young plant tissues. Polygalacturonases break bonds between polygalacturonic acid residues, thereby degrading pectin, while beta-xylosidases hydrolyse xylan, a hemicellulose component of the cell wall. It is possible that the CWDE of *E*. *fawcettii* have the ability to degrade components of a growing cell wall, however as the host cell wall matures, the *E*. *fawcettii* CWDE repertoire becomes less effective, perhaps explaining why only young plant tissues are susceptible to citrus scab. The 203 potential CWDE were also cross-referenced against PHI-base, resulting in the prioritisation of 21 proteins which had similarity to known virulence factors of plant pathogens (Table 6, S8 Table), thus highlighting candidate virulence genes of *E*. *fawcettii* for future experimental investigation. Among these 21 proteins were 14 predicted pectin-degrading enzymes, including two with similarity to polygalacturonase genes, specifically *pg1* (53.7%) and *pgx6* (66.4%) of *Fusarium oxysporum* which have been shown to reduce pathogen virulence when both are mutated simultaneously [74]; two showed similarity (61.6% and 41.8%) to the *PecA* polygalacturonase gene of *Aspergillus flavus*, a CWDE which primarily degrades pectin, and has been shown to improve pathogen invasion and increase spread during infection [73]; one with similarity to the pectin methylesterase *Bcpme1* gene of *B*. *cinerea* [78]; four with similarity (45.7% - 63.5%) to *PelA* and *PelD*, two pectate lyase virulence factors of *Nectria haematococca* [75]; and a further five obtained a pectate lyase Pfam hit, of which four showed similarity (40.3% - 53.5%) to the *Pnl1* pectin lyase gene of citrus pathogen *Penicillium digitatum* [76] and one with 58.4% similarity to *PelB* pectate lyase B gene of *Colletotrichum gloeosporioides*, seen to affect virulence on avocado [77]. A further five prioritised candidate CWDE, classed as hemicellulose-degrading enzymes, showed similarity (46.7% - 61.6%) to the endo-1,4-beta-xylanases (glycosyl hydrolase families 10 and 11) of *M*. *oryzae*, the knockdown of which is seen to reduce pathogenicity [80]. The remaining two prioritised CWDE, classed as cellulose-degrading enzymes, showed 51.9% and 52.9% similarity to the *Glu1* glucanase gene, a known virulence factor of wheat pathogen *Pyrenophora tritici-repentis* [79]. The similarities seen between these predicted secreted CAZymes and known virulence factors provides a collection of likely CWDE of *E*. *fawcettii* for future investigation. Unlike SP or CE, predicted CWDE of *E*. *fawcettii* were not overrepresented among genes found at the contig end or within 2 Kb of an AT-rich region (S8 Table). There was some crossover between CE and CWDE, with three *E*. *fawcettii* (BRIP 53147a) proteins being labelled as both prioritised CE and prioritised CWDE, thus providing some CE with potential carbohydrate-interacting functions.

**Table 6:** Predicted function of prioritised candidate cell wall degrading enzymes of *Elsinoë fawcettii*.

Gene accession	PHI-base hit	Similarity (%)	Top Pfam hit
***Predicted pectin-degrading enzymes*:**			
**KAF4548260**	PGX6 *Fusarium oxysporum* (PHI:4880)	66.39	Glycosyl hydrolases family 28 (GH28)
**KAF4556463**	PG1 *F*. *oxysporum* (PHI:4879)	53.69	GH28
**KAF4550523**	PECA *Aspergillus flavus* (PHI:88)	61.64	GH28
**KAF4547067**	PECA *A*. *flavus* (PHI:88)	41.80	GH28
**KAF4547800**	BCPME1 *Botrytis cinerea* (PHI:278)	47.97	Pectinesterase
**KAF4549166**	PelD *Nectria haematococca* (PHI:180)	47.27	Pectate lyase (PL)
**KAF4552448**	PelD *N*. *haematococca* (PHI:180)	63.45	PL
**KAF4550092**	PelA *N*. *haematococca* (PHI:179)	46.38	PL
**KAF4548090**	PelA *N*. *haematococca* (PHI:179)	45.69	PL
**KAF4549258**	PNL1 *Penicillium digitatum* (PHI:3226)	53.46	PL
**KAF4556657**	PNL1 *P*. *digitatum* (PHI:3226)	44.74	PL
**KAF4555488**	PNL1 *P*. *digitatum* (PHI:3226)	41.70	PL
**KAF4556483**	PNL1 *P*. *digitatum* (PHI:3226)	40.33	PL
**KAF4549223**	PELB *Colletotrichum gloeosporioides* (PHI:222)	58.40	PL
***Predicted Hemicellulose-degrading enzymes*:**			
**KAF4552838**	Endo-1,4-beta-xylanase *Magnaporthe oryzae* (PHI:2204)	61.56	Glycosyl hydrolase family 10 (GH10)
**KAF4550100**	Endo-1,4-beta-xylanase *M*. *oryzae* (PHI:2204)	57.69	GH10
**KAF4555167**	Endo-1,4-beta-xylanase *M*. *oryzae* (PHI:2208)	46.67	GH10
**KAF4547778**	Endo-1,4-beta-xylanase I *M*. *oryzae* (PHI:2214)	58.87	Glycosyl hydrolases family 11 (GH11)
**KAF4556368**	Endo-1,4-beta-xylanase I *M*. *oryzae* (PHI:2213)	56.72	GH11
***Predicted Cellulose-degrading enzymes*:**			
**KAF4547532**	GLU1 *Pyrenophora tritici-repentis* (PHI:3859)	52.89	Cellulase—glycosyl hydrolase family 5 (GH5)
**KAF4552889**	GLU1 *P*. *tritici-repentis* (PHI:3859)	51.93	Cellulase–GH5

### Prediction of secondary metabolite clusters

**Fig 6:**
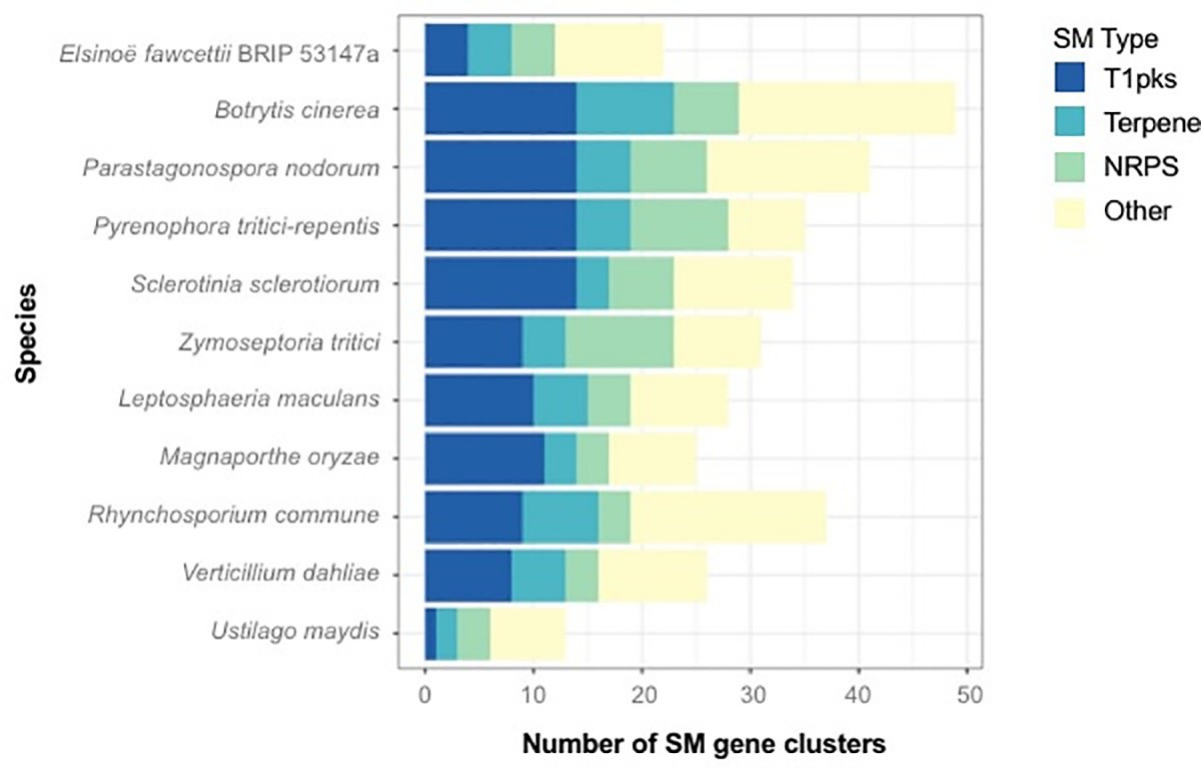
Comparison of numbers of predicted secondary metabolite gene clusters among 11 fungal species. Numbers of SM gene clusters, shown on the x axis, are divided into SM types; (I) Type I Polyketide synthase (T1PKS); (II) terpene; (III) non-ribosomal peptide synthetase (NRPS); and (IV) other, which contains all clusters identified by antiSMASH as either Type 3 Polyketide synthase (T3PKS), terpene-T1PKS, indole-T1PKS-NRPS, T1PKS-NRPS, indole-T1PKS, T1PKS-terpene-NRPS, indole, siderophore, lantipeptide, T3PKS-T1PKS or other.

Much research surrounding *E*. *fawcettii* has focused on the SM elsinochrome, which contributes to the formation of necrotic lesions [25–28]. Analysis of the *E*. *fawcettii* (BRIP 53147a) genome assembly enabled the prediction of further genes potentially involved in the elsinochrome gene cluster than previously described, as well as the prediction of additional SM clusters throughout the assembly. In total, there were 22 predicted SM clusters, involving 404 (4.0%) genes (Table 7, S9 Table). Comparing this to the results of the 10 comparative species showed that the number of predicted SM clusters varies widely among the pathogens, from 13 clusters (*U*. *maydis*) to 53 clusters (*M*. *oryzae*) (Fig 6). This wide variety among fungal species, in particular an overrepresentation of SM clusters among hemibiotrophs and necrotrophs has been seen before [160]. From the comparative analysis, it appears *E*. *fawcettii* has a lower variety of secondary metabolite clusters compared to the other necrotrophs and hemibiotrophs, particularly for Type I polyketide synthase (T1PKS) clusters. Blast analysis of the previously determined *E*. *fawcettii* elsinochrome cluster [27] against the *E*. *fawcetti* proteome indicated high similarities in amino acid sequence for six genes of the predicted T1PKS SM cluster 1 (S9 Table). Specifically, the predicted core biosynthetic gene of cluster 1 (accession KAF4556316) showed 98.6% similarity to the *E*. *fawcettii* polyketide synthase (*EfPKS1*) gene (accession ABU63483.1). An additional predicted biosynthetic gene (accession KAF4556314) had 99.6% similarity to the *E*. *fawcettii* ESC reductase (*RDT1*) gene (accession ABZ01830) and the predicted transport-related gene (accession KAF4556318) showed 70.3% similarity to the *E*. *fawcettii* ECT1 transporter (*ECT1*) gene (accession ABZ82008). Additional genes within the *E*. *fawcettii* SM cluster 1 obtained hits to the *E*. *fawcettii* elsinochrome cluster [27], specifically KAF4556317, KAF4556315 and KAF4556313 had high (97.4% - 100%) similarity to *PRF1* prefoldin protein subunit 3 (accession ABZ01833.1), *TSF1* transcription factor (accession ABZ01831.1) and *EfHP1* coding a hypothetical protein (accession ABZ82009.1). Hence, SM cluster 1 contains the two genes, *EfPKS1* and *TSF1*, which have been shown to be essential in elsinochrome production, as well as four genes (*RDT1*, *PRF1*, *ECT1* and *EfHP1*) also thought to be involved in elsinochrome biosynthesis [26, 27]. SM cluster 1 appears to lack four genes, being *OXR1*, *EfHP2*, *EfHP3* and *EfHP4*, which have all been reported to code for hypothetical proteins and not thought to be involved in biosynthesis [27]. However, to further investigate these omissions, BLAST analysis querying the nucleotide sequences of the elsinochrome cluster [27] against the contigs of the *E*. *fawcettii* genome assembly indicated regions with high similarities (99.3% - 99.7%) consistent with the location of predicted SM cluster 1 on contig SDJM01000001. The CDS of all four gene regions, however, were found to overlap with either each other or with other predicted genes. As no overlapping genes were predicted by GeneMark-ES on this isolate, it is thought the use of alternate gene model prediction programs between the studies may be a contributing factor for these differences. Further investigation through future transcriptomics analyses of *E*. *fawcettii* may provide resolution. Interestingly, SM cluster 1 consisted of an additional nine genes to the elsinochrome cluster previously described [27], all of which lay in a cluster adjacent to *ECT1*. Several of these additional genes obtained Pfam hits such as the THUMP domain, peptidase M3, Apolipoprotein O, Gar1/Naf1 RNA binding region and Endonuclease/Exonuclease/phosphatase family, suggesting these additional neighbouring proteins may perform functions such as RNA binding and modification, peptide cleavage, lipid binding and intracellular signalling, thus providing further genes for future investigation into the elsinochrome biosynthesis pathway.

**Table 7:** Predicted Secondary Metabolite (SM) gene clusters of *Elsinoë fawcettii*.

Cluster #	SM class	Genomic location (number of genes involved)	Similarity to known SM biosynthetic gene clusters		
			Known SM cluster gene (GenBank accession)	Similarity (%)	*E*. *fawcettii* GenBank accession
**1**	T1PKS	SDJM01000001, 641093:686753 (15 genes)	**Elsinochrome A/B/C:**		
			EfHP1 hypothetical protein (ABZ82009.1)	97	KAF4556313
			ESC reductase (ABZ01830.1)	100	KAF4556314
			Transcription factor (ABZ01831.1)	98	KAF4556315
			Polyketide synthase (ABU63483.1)	99	KAF4556316
			ESC prefoldin protein subunit 3 (ABZ01833.1)	100	KAF4556317
			ECT1 transporter (ABZ82008.1)	70	KAF4556318
**2**	terpene-T1PKS	SDJM01000001, 1100227:1205433 (43 genes)	**PR toxin:**		
			Short-chain dehydrogenase/reductase SDR (CDM31317.1)	54	KAF4556505
			Aristolochene synthase (CDM31315.1)	60	KAF4556513
			FAD-binding, type 2 (CDM31316.1)	42	KAF4556518
**3**	other	SDJM01000002, 204508:248496 (18 genes)			
**4**	other	SDJM01000002, 1497538:1541073 (22 genes)			
**5**	terpene	SDJM01000003, 564086:586459 (10 genes)			
**6**	terpene	SDJM01000003, 907579:930486 (11 genes)			
**7**	other	SDJM01000004, 582204:627436 (23 genes)			
**8**	other	SDJM01000006, 282237:328303 (19 genes)			
**9**	other	SDJM01000006, 329430:373960 (19 genes)			
**10**	other	SDJM01000006, 783514:830534 (17 genes)			
**11**	terpene	SDJM01000007, 20929:44027 (11 genes)			
**12**	T1PKS	SDJM01000007, 199413: 248702 (25 genes)	**Trypacidin:**		
			Putative toxin biosynthesis regulatory protein AflJ (EAL89340.1)	43	KAF4553274
			Hypothetical protein (EAL89347.1)	72	KAF4553277
			Putative metallo-beta-lactamase domain protein (EAL89338.1)	57	KAF4553279
			Putative polyketide synthase (EAL89339.1)	59	KAF4553280
			**Pestheic acid:**		
			PtaD (AGO59044.1)	57	KAF4553277
			PtaB (AGO59041.1)	63	KAF4553279
			PtaA (AGO59040.1)	59	KAF4553280
**13**	NRPS	SDJM01000008, 153859:208507 (21 genes)			
**14**	other	SDJM01000009, 468080:512558 (18 genes)			
**15**	NRPS	SDJM01000015, 163571:217225 (15 genes)			
**16**	terpene	SDJM01000020, 268495:289017 (9 genes)			
**17**	T1PKS	SDJM01000025, 66786:116804 (18 genes)			
**18**	T1PKS	SDJM01000028, 107087:155682 (17 genes)	**Cercosporin:**		
			Polyketide synthase (AAT69682.1)	53	KAF4548432
			Cercosporin toxin biosynthesis protein (ABC79591.2)	52	KAF4548433
			Oxidoreductase (ABK64184.1)	41	KAF4548434
			O-methyltransferase (ABK64180.1)	61	KAF4548436
			Oxidoreductase (ABK64182.1)	60	KAF4548439
**19**	T3PKS	SDJM01000034, 15555:58185 (18 genes)			
**20**	NRPS	SDJM01000035, 41518:95090 (22 genes)			
**21**	NRPS	SDJM01000037, 59764:106480 (19 genes)			
**22**	other	SDJM01000059, 16530:45727 (15 genes)			

An additional predicted SM cluster deserving of further investigation was SM cluster 2, a terpene-T1PKS, located 415,394 bp from the elsinochrome SM cluster 1 on contig SDJM01000001. This cluster shows sequence similarity to three proteins within the PR toxin biosynthetic gene cluster, namely aristolochene synthase (accession CDM31315.1) with 60% similarity to KAF4556513, short-chain dehydrogenase/reductase (accession CDM31317.1) with 54% similarity KAF4556505 and the type 2 FAD-binding protein (accession CDM31316.1) with 42% similarity to KAF4556518. The PR toxin is produced by the saprobe *Penicillium roqueforti*, a known contaminant of silages [161], while the mechanisms of its likely role in plant degeneration are unknown [162], PR toxin is seen to induce necrosis in human intestinal epithelial cells and monocytic immune cells [163] and exhibits mutagenic activity towards rats [164]. Thus, indicating the potential production of a toxin by *E*. *fawcettii* with DNA-binding capabilities. Another predicted SM gene cluster of interest was the T1PKS SM cluster 12. Three genes of cluster 12 (KAF4553277, KAF4553279 and KAF4553280) showed similarity to multiple known biosynthetic genes clusters; including the pestheic acid biosynthetic gene cluster of *Pestalotiopsis fici* [165] thought to function as a plant growth regulator [166] and the Trypacidin biosynthetic gene cluster of *Aspergillus fumigatus*, which produces a SM toxic to human lung cells [167]. Lastly, SM cluster 18 is predicted to code for five proteins with sequence similarity to those of the cercosporin biosynthetic gene cluster of *Cercospora nicotianae* [168]. Specifically, KAF4548432 (53% similarity to polyketide synthase, accession AAT69682.1), KAF4548433 (52% similarity to cercosporin toxin biosynthesis protein, accession ABC79591.2), KAF4548434 (41% similarity to oxidoreductase, accession ABK64184.1), KAF4548436 (61% similarity to O-methyltransferase, accession ABK64180.1) and KAF4548439 (60% similarity to oxidoreductase, accession ABK64182.1). Cercosporin, similar to elsinochrome, is a fungal toxin which promotes the generation of reactive oxygen species in the presence of light, killing plant cells [169]. Cercosporin produced by *C*. *nicotianae* has been shown to cause necrotic lesions on tobacco leaves [170] and is also produced by the apple pathogen *Colletotrichum fioriniae* [171]. While it has been shown that elsinochrome production is important for full virulence by *E*. *fawcettii* [26, 27], biosynthesis of further SMs, such as cluster 2, 12 or 18, may be beneficial to pathogenesis by potentially disrupting host plant signalling, causing additional necrosis or inhibiting competing microbes.

Analysis of the distances between predicted SM genes and TE indicated no TE were in the close vicinity of SM cluster 1 (elsinochrome), the closest TE to the edge of the cluster was 199,748 bp or 77 genes away. This lack of association was seen among all *E*. *fawcettii* predicted SM clusters, with seven clusters predicted on contigs without identified TE (S9 Table). Of those clusters which did lie on contigs with TE, genes were an average distance of 236,556 bp away, suggesting recent activity of known TE was unlikely to be involved in the formation of *E*. *fawcettii* SM clusters. The closest AT-rich region to SM cluster 1 was a distance of 90,363 bp, while this was less than the mean distance (257,863 bp), this indication of potential TE degradation by RIP is still quite distant. In contrast to multiple SP and CE seen in the close vicinity of AT-rich regions, there were no genes from predicted SM clusters within 2 Kb of an AT-rich region, suggesting genes involved in SM production may benefit from residing in more stable genomic regions.

## Conclusion

The WGS sequencing, genome mining and comparative analyses conducted in this study illustrates the potential that exists within the genome of *E*. *fawcettii* for virulence factors such as protein effectors and CWDE. The identification of these potential pathogenicity-related genes is a first step in determining further mechanisms utilised by *E*. *fawcettii* in addition to elsinochrome production, thus enabling this pathogen to defeat plant immune strategies in a host-specific manner. This study provides predicted virulence genes for future experimental investigation of *E*. *fawcettii* pathogenesis pathways, as well as establishing a comprehensive genomic resource for use in future studies to determine improved methods of control and screening of this pathogen.

## Supporting information

S1 Table GenBank accessions and genomic locations for RPB2, ITS and TEF1-α sequences included in the phylogenetic analyses with *elsinoë fawcettii* isolate (BRIP 53147a). (DOCX)

S2 Table Comparison of predicted gene classifications among *elsinoë fawcettii* and 10 other species; pfam hits, predicted CAZymes and core/unique/other genes. (XLSX)

S3 Table Sequence alignment of partial ITS and TEF1-α regions of *elsinoë fawcettii* (BRIP 53147a) in comparison with other isolates of *E*. *fawcettii* and closely related *elsinoë* species. (TXT)

S4 Table Comparison of results of EffectorP predicted candidate effectors and alternate candidate effector search among 11 species. (XLSX)

S5 Table Genomic and proteomic analyses of 15 species for use in known effector analysis and candidate effector prioritisation. (XLSX)

S6 Table Comparison of numbers of predicted secreted proteins, candidate effectors and prioritised candidate effectors among 15 species. (XLSX)

S7 Table Features and GenBank accessions of 276 *elsinoë fawcettii* BRIP 53147a candidate effectors. (XLSX)

S8 Table Features and GenBank accessions of 378 *elsinoë fawcettii* predicted CAZymes. (XLSX)

S9 Table Features and GenBank accessions of 404 *elsinoë fawcettii* genes with predicted involvement in secondary metabolite clusters. (XLSX)
