# Supplementary material for: Genome mining of the citrus pathogen Elsinoë fawcettii; prediction and prioritisation of candidate effectors, cell wall degrading enzymes and secondary metabolite gene clusters
Source: PLoS One. 2020 May 29;15(5):e0227396. doi: 10.1371/journal.pone.0227396 (PMC7259788; doi:10.1371/journal.pone.0227396)
Supplement: S1 Table — (DOCX) [file pone.0227396.s001.docx]

S1 (a) Genome assembly GenBank accessions and locations for RPB2 and TEF1-α sequences included in the phylogenetic analysis with *Elsinoë fawcettii* isolate (BRIP 53147a) in Figure 1

| Species (strain), GenBank assembly accession | Genomic location | | Reference |
| --- | --- | --- | --- |
|  | **RPB2** | **TEF1-α** |  |
| *Elsinoë fawcettii* (BRIP 53147a), SDJM00000000 | Efcl_Contig_16  566337:567078 | Accession: MN787508 |  |
| *E. fawcettii* (DAR 70024), GCA_007556565.1 | SWCR01000004.1  1491439:1492180 | SWCR01000007.1  905414:905781 | [124] |
| *E. fawcettii* (SM16-1), GCA_007556535.1 | VAAB01000009.1 527249:527992 | VAAB01000010.1  810556:810923 | [124] |
| *E. ampelina* (YL-1), GCA_005959805.1 | SMYM01000002.1  775093:775834 | SMYM01000001.1  359907:360274 | [125] |
| *E. australis* (Arg-1), GCA_007556505.1 | SWCS01000005.1 550352:551093 | SWCS01000007.1  804446:804813 | [124] |
| *Zymoseptoria tritici* (ST99CH 1E4), GCA_900184115.1 | LT854256.1  920363:921522 | LT854256.1  2059985:2060352 | [120] |
| *Parastagonospora nodorum* (SN15), GCF_000146915.1 | NW_001884567.1  399978:400673 | NW_001884568.1  56860:57227 | [117] |
| *Leptosphaeria maculans* (JN3), GCF_000230375.1 | NW_003533871.1  571201:571966 | NW_003533847.1  1591860:1592227 | [112] |
| *Pyrenophora tritici-repentis* (DW5), GCA_003231415.1 | MUXC01000023.1 96534:97465 | MUXC01000280.1  25823:26190 | [118] |
| *Rhynchosporium commune* (UK7), GCA_900074885.1 | FJUW01000001.1  941326:945224 | FJUW01000015.1  587359:587709 | [114] |
| *Botrytis cinerea* (B05.10), GCF_000143535.2 | NC_037323.1  703730:704407 | NC_037318.1  2020091:2020458 | [116] |
| *Sclerotinia sclerotiorum* (UF-70), GCF_000146945.2 | NW_001820835.1  2290220:2291017 | NW_001820830.1  1823579:1823946 | [119] |
| *Magnaporthe oryzae* (70-15), GCF_000002495.2 | NC_017844.1  6879084:6883798 | NC_017851.1  1161008:1161375 | [113] |
| *Verticillium dahlia* (Vdls.17), GCF_000150675.1 | NW_009276922.1  1110816:1115035 | NW_009276925.1  665591:665958 | [115] |
| *Ustilago maydis* (521),  GCF_000328475.2 | NC_026489.1  572250:576082 | NC_026479.1  314654:315013 | [111] |
| *Spizellomyces punctatus*, GenBank accessions for sequences | DQ302773.1 | XM_016754690.1 | [172, 173] |

S1(b). GenBank accessions for ITS and TEF1-α sequences included in the phylogenetic analysis with *Elsinoë fawcettii* isolate (BRIP 53147a) in Figure 2

| Species | ITS | TEF1-α | Reference |
| --- | --- | --- | --- |
| *Elsinoë fawcettii* BRIP 53147a | MN784182 | MN787508 |  |
| *E. fawcettii* SM3-1 | FJ010360.2 | FJ010270.2 | [3] |
| *E. fawcettii* Jin-1 | FJ010320.1 | FJ010244.1 | [3] |
| *E. fawcettii* Jin-6 | FJ010323.2 | FJ010247.2 | [3] |
| *E. fawcettii* S38162 | FJ010343.2 | FJ010267.1 | [3] |
| *E. fawcettii* DAR 70187 | FJ010290.2 | FJ010214.1 | [3] |
| *E. fawcettii* CC-132 | FJ010297.2 | FJ010222.1 | [3] |
| *E. fawcettii* CC-3 | FJ010295.1 | FJ010220.2 | [3] |
| *E. fawcettii* DAR 70024 | FJ010307.2 | FJ010231.1 | [3] |
| *E. fawcettii* CBS 139.25 | KX887219.1 | KX886865.1 | [18] |
| *E. fawcettii* CBS 231.64 | KX887220.1 | KX886866.1 | [18] |
| *E. fawcettii* CBS 232.64 | KX887221.1 | KX886867.1 | [18] |
| *E. fawcettii* CBS 233.64 | KX887222.1 | KX886868.1 | [18] |
| *E. citricola* CPC 18535 | KX887207.1 | KX886853.1 | [18] |
| *E. citricola* CPC 18570 | KX887208.1 | KX886854.1 | [18] |
| *E. pitangae* CBS 227.50 | KX887269.1 | KX886914.1 | [18] |
| *E. fagarae* CBS 514.50 | KX887218.1 | KX886864.1 | [18] |
| *E. caleae* CBS 221.50 | KX887205.1 | KX886851.1 | [18] |
| *E.* *diospyri* CBS 223.50 | KX887210.1 | KX886856.1 | [18] |
| *E.* *fici-caricae* CBS 473.62 | KX887224.1 | KX886870.1 | [18] |
| *E.* *flacourtiae* CBS 474.62 | KX887225.1 | KX886871.1 | [18] |
| *E.* *zizyphi* CBS 378.62 | KX887303.1 | KX886947.1 | [18] |
| *E.* *eucalypticola* CBS 124765 | KX887215.1 | KX886861.1 | [18] |
| *E.* *tectificae* CBS 124777 | KX887292.1 | KX886937.1 | [18] |
| *Myriangium hispanicum* CBS 247.33 | KX887304.1 | KX886948.1 | [18] |

References:

172. Lutzoni F, Kauff F, Cox CJ, McLaughlin D, Celio G, Dentinger B, Padamsee M, Hibbett D, James TY, Baloch E, Grube M. Assembling the fungal tree of life: progress, classification, and evolution of subcellular traits. American Journal of Botany. 2004 Oct;91(10):1446-80.

173. Russ C, Lang BF, Chen Z, Gujja S, Shea T, Zeng Q, Young S, Cuomo CA, Nusbaum C. Genome sequence of *Spizellomyces punctatus*. Genome Announc.. 2016 Aug 25;4(4):e00849-16.
