# Supplementary material for: Genome mining of the citrus pathogen Elsinoë fawcettii; prediction and prioritisation of candidate effectors, cell wall degrading enzymes and secondary metabolite gene clusters
Source: PLoS One. 2020 May 29;15(5):e0227396. doi: 10.1371/journal.pone.0227396 (PMC7259788; doi:10.1371/journal.pone.0227396)
Supplement: Attachment — Submitted filename: Response to Reviewers.docx [file pone.0227396.s010.docx]

| **Reviewer's comments** | **Line number/sections in updated paper (track changes version)** | **Responses** |
| --- | --- | --- |
| **Reviewer 1** |  |  |
| pages 24-34 better flow | L916 | Agree. Repetitive section removed from "Prioritisation of CEs" section |
| I could not access the raw data to assess its availability, but I assume the data will be released using the stated accession numbers following publication. | L241-244. | Yes, the data has been submitted to GenBank and will be released following publication. Once released, Table 6 & 7, L1180, L1182, L1184, L1187, L1230-1232, L1238, L1239, L1251-L1255, S7-S9 will be updated. |
| P12L278: I don’t think that categorizing genes into gene-sparse or gene-dense regions relative to the median is quite stringent enough. | L351, L353, L812, Table 3, Table 4, Fig4, S7, S8 | Gene-sparse genes changed to those with IFR > or = to 1500 bp on both sides. Gene-dense genes changed to those with IFR < 1500 bp on both sides. Amended multiple lines, Table 3, Table 4, Fig4, S7, S8. |
| P15L344: Should be 19.6 Mb? | L458 | Corrected |
| P17L376-379: Does this mean that U. maydis had the highest proportion of unique genes? | L509 | Yes, U. maydis has the highest proportion of unique genes among the comparative species. L509 amended to state this point. |
| P17L381-382: What is the rationale for this statement? Does a larger number of unique genes translate to a larger host range? | L531 | This speculative sentence has been deleted. |
| P19L422-423: Eight gene were found nearby these AT-rich regions. Are any of these predicted effectors? What are the functional domains (if any) of these genes? | L995 | Three were predicted as secreted proteins and one as a candidate effector. These eight genes were included in the "57 genes found within 2Kb of an AT-rich region" analysis for SP's and CE's. The wording of L995 has been amended to make that clear. |
| Table 2: Was Tox3 from *P. nodorum* not predicted as an effector? Was it a false negative? |  | Tox3 was predicted as a secreted protein and candidate effector. However, only known effectors that were found in the genome assemblies analysed (so that nearby genomic features could be utilised in the optimised scoring system) were included. Tox3 was not found in the GCF_000146915.1 assembly to the specifications mentioned at L344. McDonald (2013) show's *P. nodorum* isolates can have a combination of these effectors, but not necessarily all three. |
| P20L435-443: How many of these genes with repetitive sequences were classified as potential effectors? | L631-633, L982-987 | The number of candidate effectors containing PolyAA repeats or SSR's is detailed at L982-987. L631-633 has been amended to note they will be used to cross reference against CE's. |
| P22L498-502: Are there any other references in the literature to support this statement? Based on the analyses in this manuscript, only M. oryzae had a substantially larger repertoire of secreted proteins, which isn’t enough evidence to assert that hemibiotrophs produce a greater number of secreted proteins compared to necrotrophs. | L696 | This speculative sentence has been deleted. |
| P26-27L572-580: Is this increase in the presence of selected Pfam domains statistically significant? I would suggest conducting a hypergeometric test (or something similar) to test for overrepresentation of neighboring genes with these domains. | Fig3, Fig4 , Fig5, Table 2, Table 3, S5, S6, and S7. Multiple lines | Phyper and fisher tests in R have been used, only 4/10 are statistically significant. As Reviewer 2 pointed out that the Pfam criteria could be biased because of *M. oryzae*, the Pfam hits surrounding *M. oryzae* known effectors were removed and tested again. This resulted in 0/4 being statistically significant. The PFam criteria has been removed from the optimized points system and the numbers of prioritised CE's have been updated. |
| P27L588: As intergenic distances can vary greatly, I think a more appropriate analysis would compare physical distances to TEs instead of the number of genes. | Fig4, S5, S7, S9, Table 3, L851-83 | Agreed, "within 7 genes" has been changed to "within 20,000 Kb". |
| P31L609-630: This section feels like a reiteration of the previous section. I would suggest trying to condense these two sections. | L916 | Agreed. Repetitive section removed from "Prioritisation of CEs" section. |

| **Reviewer 2** |  |  |
| --- | --- | --- |
| The number of other fungal genomes used in the comparative analyses is limited. the genome sequence of a related strains and species, Elisinoe ampelina (Released 2016 by JGI) | L303-308, L458-459, L477-479, L513-544, L917, Table 2, Table 3, Fig1, Fig4, Fig5, S1, S5, S6 | This is a good point. However, while *E. ampelina* was sequenced and assembled in 2016, it wasn't publicly available until recently (Li 2020). 3x *Elsinoe* assemblies (Shumanagan 2020) also released after the paper was submitted. These 4 *Elsinoe* assemblies have been added to the paper for comparisons to BRIP 53147a. |
| One more point is that their optimized scoring system may be biased for specific genomes since the distribution of known effectors that were sampled is not even. For example, there are many more Magnaporthe ECs in their list compared to Sclerotinia. This may be an issue with the PFAM criteria used. To counter this point, maybe the authors could check for enrichment of specific PFAM domains in effectors of specific fungi vs secreted proteins in general/genes in general from specific fungi |  | See line 13 above |
| Abstract: The abstract mentions AT-rich regions. While there are certainly AT-rich regions according to the authors’ analysis with the OcculterCut program, they seem to account for only a small proportion of the genome. To better represent this nuance, it would be better to mention that in the abstract (e.g. “a small proportion of the genome is AT rich…” or something of that nature. | L42 | Amended |
| L255: It is recommended by the developers of EffectorP to run both EffectorP versions 1 and 2 since some effector proteins are better predicted by version 1 and others are better predicted by version2. I think the authors should include EffectorP v1 results | L341, L766, L1698, Table 3, Table 5, S4, S6, S7, S8 | EffectorP v1 results added to paper. |
| L174: Please include the insert size range of the paired-end libraries. | L186 | Amended L186 to include mean insert size. |
| L244: The U. maydis genome assembly is 19.6 Mb, although the estimated genome size is 20.5 Mb. | L458 | The assembly size of *U. maydis* isn't stated at L244, nor in that section (Sequence information). L458 has been amended to make it clearer that 19.6 Mb is the size of the *U. maydis* assembly that is analysed in the paper. |
| L333: Rather than using Ascomycota orthoDB, the authors should use the Dothideomycete dataset for a more accurate estimation of assembly completeness. | L208, L438 | Amended. Dothideomycetes db v10 used with BUSCO. |
| L344: The genome is much smaller than that of other fungi. Can the authors provide an estimate of the expected genome size analysis (e.g. by k-mer frequency)? In this section, the authors can provide a comparison to the already published genomes of E. fawcettii and E. australis which show similar traits in terms of size and gene number. | L207-207, L435-357, L458-459, L477-479 | Kmergenie and GenomeScope used for estimated genome size, results included in paper.Comparison to 2x *E. fawcettii* assembly sizes (Shanmugan, 2020) added. |
| P16-17: This section is a bit confusing. What is “annotated” (L356)? On the one hand, only 55.91 % of genes were “annotated” but, ~70% had a PFAM domain (L362)? |  | 55.91% genes were annotated with a gene name using the method on L231-237. 70.1% of genes had at least one hit to a Pfam model, indicating at least a section of ~70% of the genes showed some similarity to sections of already identified genes from other organisms. However, the description of some Pfam models were not so useful, for example "Protein of unknown function", "Uncharacterized conserved protein" or "Ubiquitin-2 like Rad60 SUMO-like", which was felt wouldn't add any value to annotate the genes in the way. Very few genes that obtained only a hit to a Pfam model and no hits to the Swiss-Prot Ascomycota database were annotated. |
| P17 para 2: The definition of accessory is rather broad. In general, accessory regions show absence/presence polymorphisms between closely related strains and do not affect general growth/metabolism, although they may have importance in infection. Since the fungi used in this comparative analysis are not closely related, the definition of accessory in this paper may include ascomycete-specific genes (which would be missing from U. maydis as the authors point out elsewhere) that would be still important to basal growth. It would be better to show how the proteins are conserved based on the relationship between the different fungi in this analysis in Fig. 1. To do this, the authors should add a phylogenetic tree for the fungi in Fig. 1. Genes which do not fit the expected pattern (e.g. showing a patchy conservation pattern), could be termed as “Other conservation pattern”, rather than accessory. If the authors wish to define accessory genes, they could add an analysis of genes from the other two E. fawcettii isolates, if the annotations have been made available. | Fig1,Table 2, S7 | Phylogenetic tree generated (Fig1) comparing *E. fawcettii* (BRIP 53147a), 10x comparative species from paper, 4x new *Elsinoes* and S. punctatus as outgroup, using partial TEF1-a and RPB2 genes. Tree submitted to TreeBASE (TreeBASE reviewer access: http://purl.org/phylo/treebase/phylows/study/TB2:S25890?x-access-code=dbff37c18dd9c89284c7f00f11310fde&format=html). The word "Accessory" genes changed to "other" genes on multiple lines. Addition of new table (Table 2) for ortholog classification of *Elsinoe* species. Column K added to S7 to show ortholog classification in relation to four other *Elsinoë* species/isolates. |
| L381: “A lower number…” This sentence is quite speculative given that B. cinerea shows an even lower number of unique genes but has a wide host range. Also, given the results in the section about SM clusters, another possibility is that unique genes are not well predicted in this pipeline. | L531 | This speculative sentence has been deleted. |
| L391: Do the authors mean when taking AT-rich regions out of consideration (i.e. when excluding AT-rich regions defined by OcculterCut?). | L564-565 | Sentence amended for clarity. |
| L419: Here and elsewhere. Use “end” instead of “edge” |  | Amended |
| L430: “correlate” | L615 | Amended |
| L437: Are polyAA sequences enriched in effector candidates or other genes involved in host pathogenesis? If not, then what is the basis for this statement? |  | See line 11 above. This speculative sentace has been removed. No, repeat-containing proteins were not overrepresented among the prioritised CE’s, however this analysis was included in the paper so that the point at L984-987 could be made. |
| L454: Is there a citation for the pathotype? What is this based on? | L644-645 | These are the only two pathotypes reported in Australia, L644-645 amended to make this point clear. |
| L487: Insert “,” after “Of these 1,569 proteins” |  | Amended |
| L489: Insert “,” after “secretion” |  | Amended |
| L490: “predicted” rather than “predictable” |  | Amended |
| L518: Can the authors say how many of these known effectors were used to train EffectorP version 2? If they were used in the training set, the statistics given may not reflect the true sensitivity of the program to pick up novel effectors in E. fawcettii as the authors point out. The accuracy of the program would be measured as the proportion of effectors that were not used in training, but which could be picked up by EffectorP. I think this would be useful information. | L772-L774 | Agreed. 23/45 effectors were used to train EffectorP v2. The sensitivity and specificity as reported by EffectorP v2 has been included. |
| L536: What do the authors mean by “lighter dependence”? Less genes does not equal less importance. | L797 | Agreed, amended. |
| L550: Replace “used to generate a prioritization pathway” with “used to prioritize”. I guess it is more of a “scoring system” than a “pathway” | L811 | Agreed. Amended |
| L562: “effectors” not “effector’s” | L840 | Amended |
| L577: Please test if the overrepresentation is statistically significant. |  | See line 13 above |
| L597: “species’” | L891 | Amended |
| L609-620: Repetitive of the previous section. I think it would be beneficial to cut it out. |  | See line 15 above |
| L628: Delete “revealed that” | L923 | Amended |
| L708: Adding transcriptome data would also be beneficial for the effector candidate prediction |  | Outside the scope of this paper |
| L722: Why not also compare the effector candidates to PHI base? | L1082-1086 | PHI-base hits to candidate effectors is detailed in S7, this has now been noted in the body of the paper. |
| L765: Less SM clusters predicted does not necessarily mean that SMs are not used as much in infection, only that potentially less diverse metabolites can be produced. | L1175 | Agreed, this is speculative sentence has been amended. |
| L790: 99.3-99.7% nucleotide similarity is very high. When the authors say that the genes were “degraded”, which parts were affected (e.g. intron/exon junctions, start/end codons?). On one hand, mutation of such features would indicate pseudogenization, whereas if other regions were affected, this would indicate that the prediction pipeline is probably not as sensitive as it should be and that the number of genes estimated is lower than actual, which the authors discuss in the first section. | L1200-L1205 | Agreed, appears to be difference in gene prediction, L1200-L1205 amended. |
