# Supplementary material for: Genome mining of the citrus pathogen Elsinoë fawcettii; prediction and prioritisation of candidate effectors, cell wall degrading enzymes and secondary metabolite gene clusters
Source: PLoS One. 2020 May 29;15(5):e0227396. doi: 10.1371/journal.pone.0227396 (PMC7259788; doi:10.1371/journal.pone.0227396)
Supplement: Peer review history

# Peer review: Decision Letter 0

**DOI:** 10.1371/journal.pone.0227396.r001

**Authors:** Richard A Wilson

29 Jan 2020

PONE-D-19-34838

Genome mining of the citrus pathogen Elsinoë fawcettii; prediction and prioritisation of candidate effectors, cell wall degrading enzymes and secondary metabolite gene clusters

PLOS ONE

Dear Dr. Ash,

Thank you for submitting your manuscript to PLOS ONE. After careful consideration, we feel that it has merit but does not fully meet PLOS ONE’s publication criteria as it currently stands. Therefore, we invite you to submit a revised version of the manuscript that addresses the points raised during the review process.

This was an interesting study on an important topic. Both reviewers recognized that the work was well done and thorough, and that the results are a great resource for the research community. However, some areas for improvement were noted. In particular: Reviewer 1 indicated that the flow of the paper could be improved. Reviewer 2 noted that the number of fungal genomes used in the comparison was limited while other Elisinoe genomes are available that might be included. Reviewer 2 also had concerns that the optimized scoring system may be biased. Please endeavor to address these specific concerns and all reviewer comments.

We would appreciate receiving your revised manuscript by Mar 14 2020 11:59PM. When you are ready to submit your revision, log on to https://www.editorialmanager.com/pone/ and select the 'Submissions Needing Revision' folder to locate your manuscript file.

If you would like to make changes to your financial disclosure, please include your updated statement in your cover letter.

To enhance the reproducibility of your results, we recommend that if applicable you deposit your laboratory protocols in protocols.io, where a protocol can be assigned its own identifier (DOI) such that it can be cited independently in the future. For instructions see: http://journals.plos.org/plosone/s/submission-guidelines#loc-laboratory-protocols

Please include the following items when submitting your revised manuscript:

- A rebuttal letter that responds to each point raised by the academic editor and reviewer(s). This letter should be uploaded as separate file and labeled 'Response to Reviewers'.
- A marked-up copy of your manuscript that highlights changes made to the original version. This file should be uploaded as separate file and labeled 'Revised Manuscript with Track Changes'.
- An unmarked version of your revised paper without tracked changes. This file should be uploaded as separate file and labeled 'Manuscript'.

Please note while forming your response, if your article is accepted, you may have the opportunity to make the peer review history publicly available. The record will include editor decision letters (with reviews) and your responses to reviewer comments. If eligible, we will contact you to opt in or out.

We look forward to receiving your revised manuscript.

Kind regards,

Richard A Wilson

Academic Editor

PLOS ONE

Journal Requirements:

1. When submitting your revision, we need you to address these additional requirements.

Please ensure that your manuscript meets PLOS ONE's style requirements, including those for file naming. The PLOS ONE style templates can be found at

http://www.journals.plos.org/plosone/s/file?id=wjVg/PLOSOne_formatting_sample_main_body.pdf and http://www.journals.plos.org/plosone/s/file?id=ba62/PLOSOne_formatting_sample_title_authors_affiliations.pdf

[Note: HTML markup is below. Please do not edit.]

Reviewers' comments:

Reviewer's Responses to Questions

**Comments to the Author**

1. Is the manuscript technically sound, and do the data support the conclusions?

The manuscript must describe a technically sound piece of scientific research with data that supports the conclusions. Experiments must have been conducted rigorously, with appropriate controls, replication, and sample sizes. The conclusions must be drawn appropriately based on the data presented. 

Reviewer #1: Yes

Reviewer #2: Partly

**********

2. Has the statistical analysis been performed appropriately and rigorously? 

Reviewer #1: Yes

Reviewer #2: N/A

**********

3. Have the authors made all data underlying the findings in their manuscript fully available?

The PLOS Data policy requires authors to make all data underlying the findings described in their manuscript fully available without restriction, with rare exception (please refer to the Data Availability Statement in the manuscript PDF file). The data should be provided as part of the manuscript or its supporting information, or deposited to a public repository. For example, in addition to summary statistics, the data points behind means, medians and variance measures should be available. If there are restrictions on publicly sharing data—e.g. participant privacy or use of data from a third party—those must be specified.

Reviewer #1: Yes

Reviewer #2: Yes

**********

4. Is the manuscript presented in an intelligible fashion and written in standard English?

PLOS ONE does not copyedit accepted manuscripts, so the language in submitted articles must be clear, correct, and unambiguous. Any typographical or grammatical errors should be corrected at revision, so please note any specific errors here.

Reviewer #1: Yes

Reviewer #2: Yes

**********

5. Review Comments to the Author

Please use the space provided to explain your answers to the questions above. You may also include additional comments for the author, including concerns about dual publication, research ethics, or publication ethics. (Please upload your review as an attachment if it exceeds 20,000 characters)

Reviewer #1: Jeffress et al. present the sequencing and genomic analysis of the important citrus pathogen Elsinoe fawcetii. Annotation of the de novo assembled genome resulted in the identification of a multitude of secreted proteins, predicted effector proteins, cell wall degrading enzymes, and secondary metabolite clusters. A prioritization pipeline highlighted 77 genes as candidate E. fawcetii effectors. These results present a great resource for the research community. Overall, the work is well done and the analyses conducted were very thorough. The manuscript contains a lot of information and I would encourage the authors to improve the flow of the paper. Particularly pages 24-34 could use some polishing. At times, the sections seemed to be slightly repetitive and could be condensed or streamlined. I could not access the raw data to assess its availability, but I assume the data will be released using the stated accession numbers following publication. Outlined below are some minor suggestions that may help improve the manuscript:

P12L278: I don’t think that categorizing genes into gene-sparse or gene-dense regions relative to the median is quite stringent enough. Making this assumption may lead to false inferences and negatively affect the prioritization of effector candidates. What does the distribution of IFR distances look like? Examination and description of this distribution would be more informative. Redoing this analysis will alter the classification of effector candidates, however, I believe it will make the analysis more robust. Alternatively, the authors could illustrate IFR distance in a similar manner to Haas et al. (2009, https://doi.org/10.1038/nature08358)

P15L344: Should be 19.6 Mb?

P17L376-379: Does this mean that U. maydis had the highest proportion of unique genes?

P17L381-382: What is the rationale for this statement? Does a larger number of unique genes translate to a larger host range?

P19L422-423: Eight gene were found nearby these AT-rich regions. Are any of these predicted effectors? What are the functional domains (if any) of these genes?

Table 2: Was Tox3 from P. nodorum not predicted as an effector? Was it a false negative?

P20L435-443: How many of these genes with repetitive sequences were classified as potential effectors?

P22L498-502: Are there any other references in the literature to support this statement? Based on the analyses in this manuscript, only M. oryzae had a substantially larger repertoire of secreted proteins, which isn’t enough evidence to assert that hemibiotrophs produce a greater number of secreted proteins compared to necrotrophs.

P26-27L572-580: Is this increase in the presence of selected Pfam domains statistically significant? I would suggest conducting a hypergeometric test (or something similar) to test for overrepresentation of neighboring genes with these domains.

P27L588: As intergenic distances can vary greatly, I think a more appropriate analysis would compare physical distances to TEs instead of the number of genes.

P31L609-630: This section feels like a reiteration of the previous section. I would suggest trying to condense these two sections.

Reviewer #2: The authors performed whole genome sequencing of Elsinoë fawcettii, an agriculturally significant fungus that infects citrus plants. They annotated the genome and identified several classes of pathogenicity-related genes including effectors, CAzymes and secondary metabolites. They also developed an additional scoring system to prioritize effector candidates for further analysis based on additional genomic traits. The analyses presented are mostly sound, however, the number of other fungal genomes used in the comparative analyses is limited. Furthermore, the genome sequence of a related strains and species, Elisinoe ampelina (Released 2016 by JGI), Elsinoë fawcettii and Elsinoë australis (https://doi.org/10.1094/MPMI-06-19-0169-A, First look: 2 Oct 2019) are available, which would be better to include if possible, and is not mentioned in the text. One more point is that their optimized scoring system may be biased for specific genomes since the distribution of known effectors that were sampled is not even. For example, there are many more Magnaporthe ECs in their list compared to Sclerotinia. This may be an issue with the PFAM criteria used. To counter this point, maybe the authors could check for enrichment of specific PFAM domains in effectors of specific fungi vs secreted proteins in general/genes in general from specific fungi. Also, some of the conclusions/discussion points are speculative.

Specific points:

Abstract: The abstract mentions AT-rich regions. While there are certainly AT-rich regions according to the authors’ analysis with the OcculterCut program, they seem to account for only a small proportion of the genome. To better represent this nuance, it would be better to mention that in the abstract (e.g. “a small proportion of the genome is AT rich…” or something of that nature.

L255: It is recommended by the developers of EffectorP to run both EffectorP versions 1 and 2 since some effector proteins are better predicted by version 1 and others are better predicted by version2. I think the authors should include EffectorP v1 results

L174: Please include the insert size range of the paired-end libraries.

L244: The U. maydis genome assembly is 19.6 Mb, although the estimated genome size is 20.5 Mb.

L333: Rather than using Ascomycota orthoDB, the authors should use the Dothideomycete dataset for a more accurate estimation of assembly completeness.

L344: The genome is much smaller than that of other fungi. Can the authors provide an estimate of the expected genome size analysis (e.g. by k-mer frequency)? In this section, the authors can provide a comparison to the already published genomes of E. fawcettii and E. australis which show similar traits in terms of size and gene number.

P16-17: This section is a bit confusing. What is “annotated” (L356)? On the one hand, only 55.91 % of genes were “annotated” but, ~70% had a PFAM domain (L362)?

P17 para 2: The definition of accessory is rather broad. In general, accessory regions show absence/presence polymorphisms between closely related strains and do not affect general growth/metabolism, although they may have importance in infection. Since the fungi used in this comparative analysis are not closely related, the definition of accessory in this paper may include ascomycete-specific genes (which would be missing from U. maydis as the authors point out elsewhere) that would be still important to basal growth. It would be better to show how the proteins are conserved based on the relationship between the different fungi in this analysis in Fig. 1. To do this, the authors should add a phylogenetic tree for the fungi in Fig. 1. Genes which do not fit the expected pattern (e.g. showing a patchy conservation pattern), could be termed as “Other conservation pattern”, rather than accessory. If the authors wish to define accessory genes, they could add an analysis of genes from the other two E. fawcettii isolates, if the annotations have been made available.

L381: “A lower number…” This sentence is quite speculative given that B. cinerea shows an even lower number of unique genes but has a wide host range. Also, given the results in the section about SM clusters, another possibility is that unique genes are not well predicted in this pipeline.

L391: Do the authors mean when taking AT-rich regions out of consideration (i.e. when excluding AT-rich regions defined by OcculterCut?).

L419: Here and elsewhere. Use “end” instead of “edge”

L430: “correlate”

L437: Are polyAA sequences enriched in effector candidates or other genes involved in host pathogenesis? If not, then what is the basis for this statement?

L454: Is there a citation for the pathotype? What is this based on?

L487: Insert “,” after “Of these 1,569 proteins”

L489: Insert “,” after “secretion”

L490: “predicted” rather than “predictable”

L518: Can the authors say how many of these known effectors were used to train EffectorP version 2? If they were used in the training set, the statistics given may not reflect the true sensitivity of the program to pick up novel effectors in E. fawcettii as the authors point out. The accuracy of the program would be measured as the proportion of effectors that were not used in training, but which could be picked up by EffectorP. I think this would be useful information.

L536: What do the authors mean by “lighter dependence”? Less genes does not equal less importance.

L550: Replace “used to generate a prioritization pathway” with “used to prioritize”. I guess it is more of a “scoring system” than a “pathway”

L562: “effectors” not “effector’s”

L577: Please test if the overrepresentation is statistically significant.

L597: “species’”

L609-620: Repetitive of the previous section. I think it would be beneficial to cut it out.

L628: Delete “revealed that”

L708: Adding transcriptome data would also be beneficial for the effector candidate prediction

L722: Why not also compare the effector candidates to PHI base?

L765: Less SM clusters predicted does not necessarily mean that SMs are not used as much in infection, only that potentially less diverse metabolites can be produced.

L790: 99.3-99.7% nucleotide similarity is very high. When the authors say that the genes were “degraded”, which parts were affected (e.g. intron/exon junctions, start/end codons?). On one hand, mutation of such features would indicate pseudogenization, whereas if other regions were affected, this would indicate that the prediction pipeline is probably not as sensitive as it should be and that the number of genes estimated is lower than actual, which the authors discuss in the first section.

**********

6. PLOS authors have the option to publish the peer review history of their article (what does this mean?). If published, this will include your full peer review and any attached files.

If you choose “no”, your identity will remain anonymous but your review may still be made public.

**Do you want your identity to be public for this peer review?** For information about this choice, including consent withdrawal, please see our Privacy Policy.

Reviewer #1: No

Reviewer #2: No

[NOTE: If reviewer comments were submitted as an attachment file, they will be attached to this email and accessible via the submission site. Please log into your account, locate the manuscript record, and check for the action link "View Attachments". If this link does not appear, there are no attachment files to be viewed.]

While revising your submission, please upload your figure files to the Preflight Analysis and Conversion Engine (PACE) digital diagnostic tool, https://pacev2.apexcovantage.com/. PACE helps ensure that figures meet PLOS requirements. To use PACE, you must first register as a user. Registration is free. Then, login and navigate to the UPLOAD tab, where you will find detailed instructions on how to use the tool. If you encounter any issues or have any questions when using PACE, please email us at figures@plos.org. Please note that Supporting Information files do not need this step.

# Author comment: Author response to Decision Letter 0

**DOI:** 10.1371/journal.pone.0227396.r002

11 Mar 2020

The responses have been included in the file "Response to reviewers doc" as attached to the submission. The response shows detailed changes made to the document as requested.

Attachment Submitted filename: Response to Reviewers.docx Click here for additional data file.

# Peer review: Decision Letter 1

**DOI:** 10.1371/journal.pone.0227396.r003

**Authors:** Richard A Wilson

31 Mar 2020

PONE-D-19-34838R1

Genome mining of the citrus pathogen Elsinoë fawcettii; prediction and prioritisation of candidate effectors, cell wall degrading enzymes and secondary metabolite gene clusters

PLOS ONE

Dear Dr. Ash,

Thank you for submitting your manuscript to PLOS ONE. After careful consideration, we feel that it has merit but does not fully meet PLOS ONE’s publication criteria as it currently stands. Therefore, we invite you to submit a revised version of the manuscript that addresses the points raised during the review process.

Both reviewer's noted most comments had been addressed and the manuscript is much improved. A few additional comments could be considered, in particular the missing figures should be addressed.

We would appreciate receiving your revised manuscript by May 15 2020 11:59PM. When you are ready to submit your revision, log on to https://www.editorialmanager.com/pone/ and select the 'Submissions Needing Revision' folder to locate your manuscript file.

If you would like to make changes to your financial disclosure, please include your updated statement in your cover letter.

To enhance the reproducibility of your results, we recommend that if applicable you deposit your laboratory protocols in protocols.io, where a protocol can be assigned its own identifier (DOI) such that it can be cited independently in the future. For instructions see: http://journals.plos.org/plosone/s/submission-guidelines#loc-laboratory-protocols

Please include the following items when submitting your revised manuscript:

- A rebuttal letter that responds to each point raised by the academic editor and reviewer(s). This letter should be uploaded as separate file and labeled 'Response to Reviewers'.
- A marked-up copy of your manuscript that highlights changes made to the original version. This file should be uploaded as separate file and labeled 'Revised Manuscript with Track Changes'.
- An unmarked version of your revised paper without tracked changes. This file should be uploaded as separate file and labeled 'Manuscript'.

Please note while forming your response, if your article is accepted, you may have the opportunity to make the peer review history publicly available. The record will include editor decision letters (with reviews) and your responses to reviewer comments. If eligible, we will contact you to opt in or out.

We look forward to receiving your revised manuscript.

Kind regards,

Richard A Wilson

Academic Editor

PLOS ONE

[Note: HTML markup is below. Please do not edit.]

Reviewers' comments:

Reviewer's Responses to Questions

**Comments to the Author**

1. If the authors have adequately addressed your comments raised in a previous round of review and you feel that this manuscript is now acceptable for publication, you may indicate that here to bypass the “Comments to the Author” section, enter your conflict of interest statement in the “Confidential to Editor” section, and submit your "Accept" recommendation.

Reviewer #1: (No Response)

Reviewer #2: (No Response)

**********

2. Is the manuscript technically sound, and do the data support the conclusions?

The manuscript must describe a technically sound piece of scientific research with data that supports the conclusions. Experiments must have been conducted rigorously, with appropriate controls, replication, and sample sizes. The conclusions must be drawn appropriately based on the data presented. 

Reviewer #1: Yes

Reviewer #2: Yes

**********

3. Has the statistical analysis been performed appropriately and rigorously? 

Reviewer #1: Yes

Reviewer #2: Yes

**********

4. Have the authors made all data underlying the findings in their manuscript fully available?

The PLOS Data policy requires authors to make all data underlying the findings described in their manuscript fully available without restriction, with rare exception (please refer to the Data Availability Statement in the manuscript PDF file). The data should be provided as part of the manuscript or its supporting information, or deposited to a public repository. For example, in addition to summary statistics, the data points behind means, medians and variance measures should be available. If there are restrictions on publicly sharing data—e.g. participant privacy or use of data from a third party—those must be specified.

Reviewer #1: Yes

Reviewer #2: No

**********

5. Is the manuscript presented in an intelligible fashion and written in standard English?

PLOS ONE does not copyedit accepted manuscripts, so the language in submitted articles must be clear, correct, and unambiguous. Any typographical or grammatical errors should be corrected at revision, so please note any specific errors here.

Reviewer #1: Yes

Reviewer #2: Yes

**********

6. Review Comments to the Author

Please use the space provided to explain your answers to the questions above. You may also include additional comments for the author, including concerns about dual publication, research ethics, or publication ethics. (Please upload your review as an attachment if it exceeds 20,000 characters)

Reviewer #1: Jeffress et al. submitted a revision to a manuscript describing the genome sequencing of Elsinoe fawcettii, as well as comparative genomics analyses to other fungal species and effector prioritization. The authors have made nearly all of the revisions suggested by the reviewers. Overall, the manuscript has been much improved. I have a few minor suggestions for the further clarification of some aspects of the manuscript.

P9L200: capitalize Dothideomycetes

P9L201: Since mitochondrial sequences were used in this analysis, which typically are AT-rich, I suggest the authors attempt to determine what proportion of identified AT-rich regions are nuclear or mitochondrial. Alternatively, the analysis could be conducted again without mitochondrial contigs.

P10L229: I think it would be useful to mention the specific species that you are using in the comparative/phylogenetic analyses here.

P15L352: capitalize Dothideomycetes

Figure 2: I wasn’t able to view this figure. I’m not sure if it wasn’t uploaded with the manuscript or I missed it, but I can’t seem to find it. Same with S3.

P27L572: A quick sentence or two discussing the differences between EffectorP versions 1 and 2 might be useful here.

P28L613: Is this supposed to be 20,000 bp (i.e. 20 kb)?

P28L614: Is this supposed to be 20,000 bp (i.e. 20 kb)?

P35L675-676: Are these significantly different? All of these comparisons of proportions should be tested.

P40L783: Define T1PKS before abbreviating (it is defined later).

P41L809: change to “investigation”

Reviewer #2: I think the analysis is much improved with the addition of the additional genome sequences and comparisons to these other Elsinoe genomes and the omission of the PFAM domains of neighboring genes as a criteria. Here are a few minor suggestions:

L276: I think the authors mean “the EffectorP 2.0 study” rather than “EffectorP 2.0”

L306: Do the authors mean “grouping proteins into ortholog groups” rather than “mapping proteins into ortholog groups”?

L310: “compared species” rather than “comparative species”

No need for apostrophes after SP’s and CE’s

L660: I think it’s 20 kb? Not 20,000 kb?

The Treebase matrix was not available. I could only access the accession numbers of the taxa.

**********

7. PLOS authors have the option to publish the peer review history of their article (what does this mean?). If published, this will include your full peer review and any attached files.

If you choose “no”, your identity will remain anonymous but your review may still be made public.

**Do you want your identity to be public for this peer review?** For information about this choice, including consent withdrawal, please see our Privacy Policy.

Reviewer #1: No

Reviewer #2: No

[NOTE: If reviewer comments were submitted as an attachment file, they will be attached to this email and accessible via the submission site. Please log into your account, locate the manuscript record, and check for the action link "View Attachments". If this link does not appear, there are no attachment files to be viewed.]

While revising your submission, please upload your figure files to the Preflight Analysis and Conversion Engine (PACE) digital diagnostic tool, https://pacev2.apexcovantage.com/. PACE helps ensure that figures meet PLOS requirements. To use PACE, you must first register as a user. Registration is free. Then, login and navigate to the UPLOAD tab, where you will find detailed instructions on how to use the tool. If you encounter any issues or have any questions when using PACE, please email us at figures@plos.org. Please note that Supporting Information files do not need this step.

# Author comment: Author response to Decision Letter 1

**DOI:** 10.1371/journal.pone.0227396.r004

16 Apr 2020

See attached point-by-point response

Attachment Submitted filename: Second Response to reviewers comments 160420.xlsx Click here for additional data file.

# Editor report: Decision Letter 2

**DOI:** 10.1371/journal.pone.0227396.r005

**Authors:** Richard A Wilson

20 Apr 2020

Genome mining of the citrus pathogen Elsinoë fawcettii; prediction and prioritisation of candidate effectors, cell wall degrading enzymes and secondary metabolite gene clusters

PONE-D-19-34838R2

Dear Dr. Ash,

We are pleased to inform you that your manuscript has been judged scientifically suitable for publication and will be formally accepted for publication once it complies with all outstanding technical requirements.

Within one week, you will receive an e-mail containing information on the amendments required prior to publication. When all required modifications have been addressed, you will receive a formal acceptance letter and your manuscript will proceed to our production department and be scheduled for publication.

Shortly after the formal acceptance letter is sent, an invoice for payment will follow. To ensure an efficient production and billing process, please log into Editorial Manager at https://www.editorialmanager.com/pone/, click the "Update My Information" link at the top of the page, and update your user information. If you have any billing related questions, please contact our Author Billing department directly at authorbilling@plos.org.

If your institution or institutions have a press office, please notify them about your upcoming paper to enable them to help maximize its impact. If they will be preparing press materials for this manuscript, you must inform our press team as soon as possible and no later than 48 hours after receiving the formal acceptance. Your manuscript will remain under strict press embargo until 2 pm Eastern Time on the date of publication. For more information, please contact onepress@plos.org.

With kind regards,

Richard A Wilson

Academic Editor

PLOS ONE

Additional Editor Comments (optional):

Reviewers' comments:

# Editor report: Acceptance letter

**DOI:** 10.1371/journal.pone.0227396.r006

**Authors:** Richard A Wilson

29 Apr 2020

PONE-D-19-34838R2 

Genome mining of the citrus pathogen Elsinoë fawcettii; prediction and prioritisation of candidate effectors, cell wall degrading enzymes and secondary metabolite gene clusters 

Dear Dr. Ash:

I am pleased to inform you that your manuscript has been deemed suitable for publication in PLOS ONE. Congratulations! Your manuscript is now with our production department. 

If your institution or institutions have a press office, please notify them about your upcoming paper at this point, to enable them to help maximize its impact. If they will be preparing press materials for this manuscript, please inform our press team within the next 48 hours. Your manuscript will remain under strict press embargo until 2 pm Eastern Time on the date of publication. For more information please contact onepress@plos.org.

For any other questions or concerns, please email plosone@plos.org. 

Thank you for submitting your work to PLOS ONE.

With kind regards,

PLOS ONE Editorial Office Staff

on behalf of

Dr. Richard A Wilson 

Academic Editor

PLOS ONE
